# Reaction dynamics of the chimeric channelrhodopsin C1C2

**DOI:** 10.1038/s41598-017-07363-w

**Published:** 2017-08-03

**Authors:** Yusaku Hontani, Marco Marazzi, Katja Stehfest, Tilo Mathes, Ivo H. M. van Stokkum, Marcus Elstner, Peter Hegemann, John T. M. Kennis

**Affiliations:** 1 Department of Physics and Astronomy, Vrije Universiteit Amsterdam, De Boelelaan 1081, 1081 HV Amsterdam, The Netherlands; 2 Institute of Physical Chemistry, Karlsruhe Institute of Technology, Karlstrasse 12, D-76131 Karlsruhe, Germany; 3 Unidad Docente de Química Física, Universidad de Alcalá, E-28871 Alcalá de Henares, Madrid Spain; 4 Institute of Biology, Experimental Biophysics, Humboldt-Universität, Invalidenstraße 42, D-10115 Berlin, Germany; 5 Théorie-Modélisation-Simulation, Université de Lorraine-Nancy & CNRS, SRSMC, Boulevard des Aiguillettes, Vandoeuvre-lès-Nancy, France

## Abstract

Channelrhodopsin (ChR) is a key protein of the optogenetic toolkit. C1C2, a functional chimeric protein of *Chlamydomonas reinhardtii* ChR1 and ChR2, is the only ChR whose crystal structure has been solved, and thus uniquely suitable for structure-based analysis. We report C1C2 photoreaction dynamics with ultrafast transient absorption and multi-pulse spectroscopy combined with target analysis and structure-based hybrid quantum mechanics/molecular mechanics calculations. Two relaxation pathways exist on the excited (S_1_) state through two conical intersections CI_1_ and CI_2_, that are reached via clockwise and counter-clockwise rotations: (i) the C13=C14 isomerization path with 450 fs via CI_1_ and (ii) a relaxation path to the initial ground state with 2.0 ps and 11 ps via CI_2_, depending on the hydrogen-bonding network, hence indicating active-site structural heterogeneity. The presence of the additional conical intersection CI_2_ rationalizes the relatively low quantum yield of photoisomerization (30 ± 3%), reported here. Furthermore, we show the photoreaction dynamics from picoseconds to seconds, characterizing the complete photocycle of C1C2.

## Introduction

The discovery of channelrhodopsins (ChRs)^1, 2^ lead to a breakthrough in optogenetics^3^, which became a key technology in current neuroscience to optically control neural activity with high spatio-temporal precision^4–6^. ChRs are light-gated cation channels first discovered in *Chlamydomonas reinhardtii* ^1, 2^, employing all-*trans* retinal as a chromophore. So far, diverse ChR variants have been engineered for customized optogenetic applications regarding excitability/color tuning^7, 8^, ion conductance^9–11^ and modulation of the open state lifetime^12^. For rational design of further functional ChRs, clarification of the photoreaction mechanisms is highly required, yet the understanding of the molecular dynamics remains to a significant extent elusive. Photoisomerization from all-*trans* to 13-*cis* retinal, which is a key reaction to trigger the photocycle and also decides the quantum yield of the photoreaction, occurs in the femto- to picosecond time region. On the other hand, the channel pore is formed and opened (on-gating) in the microsecond/millisecond timescale following several photoinitiated intermediate states. Therefore, observation of the molecular dynamics from the femtosecond to millisecond time scales is essential for elucidation of the activation mechanisms of ChRs.

**Figure 1:**
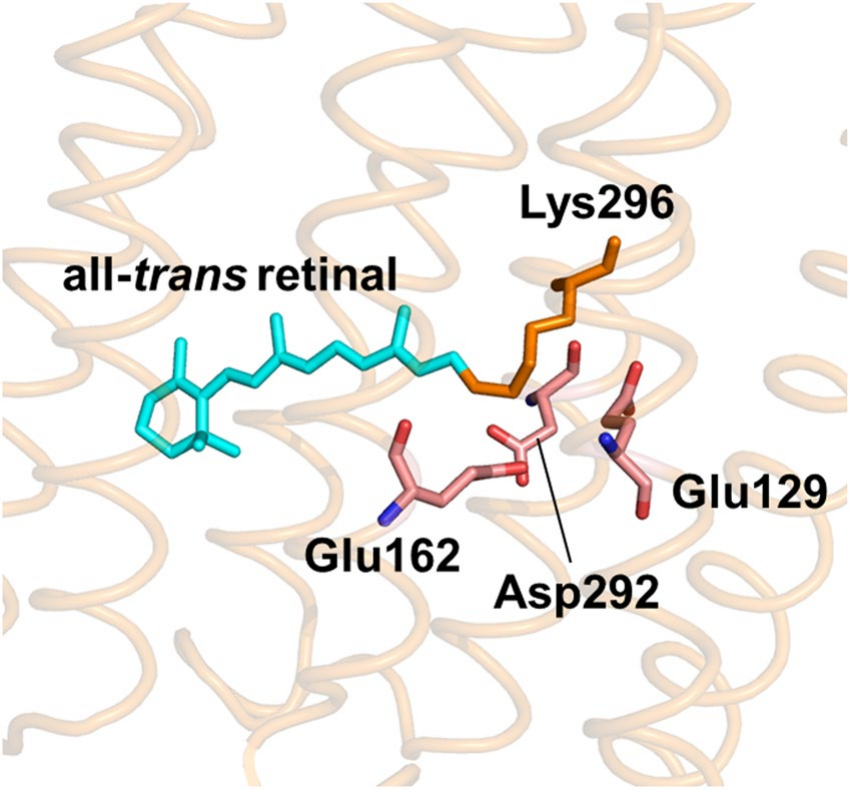
Close-up of the retinal binding pocket of C1C2^13^ (PDB ID: 3UG9).

A combination of time-resolved information on photoactivation with structure-based calculations is a powerful approach to obtain a molecular picture of the photoinduced initial structural dynamics of ChRs. C1C2, a chimeric protein of *Chlamydomonas reinhardtii* ChR1 and *Chlamydomonas reinhardtii* ChR2, is the only channelrhodopsin whose crystal structures were solved up to now^13, 14^, providing the first direct molecular basis to understand the activation process of ChRs (Fig. 1). C1C2 consists of five transmembrane helices (TM1–TM5) derived from ChR1 and two helices (TM6 and TM7) from ChR2. While several ultrafast time-resolved spectroscopies of ChR2 were performed^15–17^, no time-resolved spectroscopic data for C1C2 has been reported so far, which has restricted a systematic combination of experiments with computational calculation. Importantly, it has recently become clear that the dark-state structure in the vicinity of the retinal chromophore differs significantly between C1C2 and ChR2. In C1C2, the protonated retinal Schiff base forms a hydrogen-bond with Glu162, but not with Asp292^18^. On the other hand, in ChR2, the retinal Schiff base forms hydrogen-bonds to both of Glu123 (Glu162 in C1C2) and Asp253 (Asp292 in C1C2) and also to possibly water molecules^19^. The dark-state structural difference would result in different photoreaction dynamics in C1C2 and ChR2, thus the time-resolved experimental results of C1C2 are indispensable for direct access to theoretical approach based on the C1C2 structure.

Here we report a comprehensive femtosecond-to-second transient absorption study of C1C2. In combination with pump-dump-probe spectroscopy, target analysis and structure-based hybrid quantum mechanics/molecular mechanics calculations, we propose a multi-path excited-state dynamics model on C1C2 with two distinct conical intersections; a 450 fs isomerization path and a relaxation path with 2.0 ps and 11 ps. We further present a complete photocycle model of C1C2 from femtoseconds to seconds, which will be beneficial for further understanding of the activation mechanism of ChRs, and reveal striking kinetic differences on ground state dynamics between C1C2 and ChR2.

## Results and Discussion

**Figure 2:**
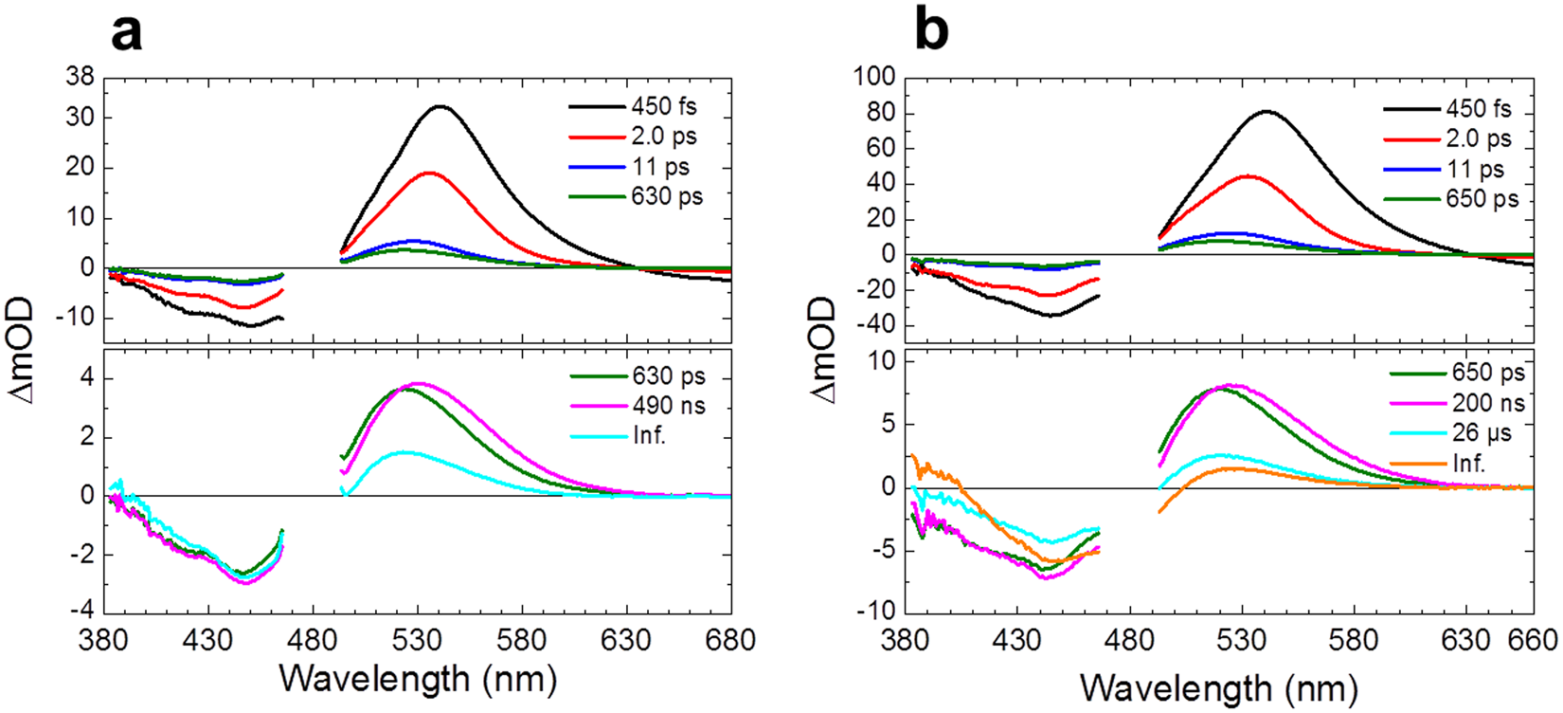
Globally fitted transient absorption spectra of C1C2. EADS of transient absorption data of C1C2 at (**a**) pH 8 and (**b**) pH 10. At pH 8, the spectra were fitted with six components, while seven components were required for pH 10 data. The spectral region of 465–495 nm is omitted because of the strong pump scattering. The 630 ps (at pH 8) and 650 ps (at pH 10) components are shown on both of the top and the bottom panels for reference.

Fig. 2 shows the evolution-associated difference spectra (EADS) determined by global analysis of the transient absorption data of C1C2 at pH 8 (Fig. 2a) and pH 10 (Fig. 2b). Six (pH 8) and seven (pH 10) spectral components were required to adequately represent the experimental data with the sequential time constants: 450 fs, 2.0 ps, 11 ps, 630 ps, 490 ns and infinity (pH 8); 450 fs, 2.0 ps, 11 ps, 650 ps, 200 ns, 26 μs and infinity (pH 10). The top panels show the early time evolution up to 11 ps, the lower panels the later stages of the evolution from 11 ps onwards. No significant differences were observed between the transient absorption spectra in H_2_O and D_2_O up to 100 μs (Figs 2 and S2), *i.e*. H/D kinetics isotope effects are not significant in the reactions. Fig. S3 shows raw kinetic traces over the entire spectral range, along with the result of the global fit. Fig. S4 shows the decay-associated difference spectra (DADS) that follow from a parallel, sum-of-exponential analysis of the data, which is mathematically equivalent to a sequential analysis. The analysis program calculates both EADS and DADS and the time constants that follow from the analysis apply to both^20^.

### Primary photochemistry: ultrafast transient absorption spectroscopy

We first consider the primary reactions that take place on the femto- to picosecond timescale shown in the upper panel of Fig. 2a (pH 8). The positive signals of the EADS consist of excited state absorption (ESA) and/or absorption of early photoproducts. The negative signals at around 460 nm is due to ground state bleach. In the 450 fs EADS, the large positive band near 540 nm may be assigned to ESA, in line with earlier work on ChR2^15, 16^. The negative signals at wavelengths >630 nm occur in a region without ground state absorption and hence must be assigned to stimulated emission (SE), providing clear information on the excited state dynamics. The SE signals decayed with 450 fs, 2.0 ps and 11 ps as highlighted in the kinetic trace at 750 nm in Fig. S5 (from pump-dump-probe experiments that are described and discussed in detail below), which implies that the excited-state decay occurs with those three time components. The excited-state absorption peaked at 541 nm immediately after the photon absorption (black line), and progressively blue-shifted to 535 nm in 450 fs (red line), and 528 nm in 2.0 ps (blue line), along with a spectral narrowing and a blue-shift of the isosbestic point. Such narrowing and blue-shifting is similar to that observed in protonated Schiff base retinal in solution, and may be assigned to vibrational relaxation and evolution on the excited-state potential energy surface^21^. Because the SE completely decayed in 11 ps, the 522-nm absorption that was left after the 11 ps evolution (green line) is only composed of the primary ground-state photoproduct, in addition to ground-state bleach of the reactant around 450 nm. No significant difference was found on the femto- to picosecond dynamics between pH 8 and 10.

### Primary photochemistry: pump-dump-probe spectroscopy

As seen in Fig. 2, the spectra of ESA and photoproduct absorption are strongly overlapped around 530 nm, which makes it difficult to distinguish multiphasic excited-state decay and vibrational relaxation from the primary product formation, and possible relaxation phenomena of the latter. This situation is similar to that observed in ChR2^15^, but differs from bacteriorhodopsin, where the ESA is blue-shifted from the ground-state bleach^22^ and the red-shifted primary photoproduct J/K is comparatively cleanly observed. To assess primary photodynamics in more detail, we applied pump-dump-probe spectroscopy to C1C2. Pump-dump-probe, or multi-pulse spectroscopy, is a powerful technique to disentangle complex photoreaction dynamics^23–33^. This technique can be used to control and influence the course of reactions as they evolve, and its power lies in the ability to selectively remove or transfer the population of transient species with carefully timed laser pulses.

**Figure 3:**
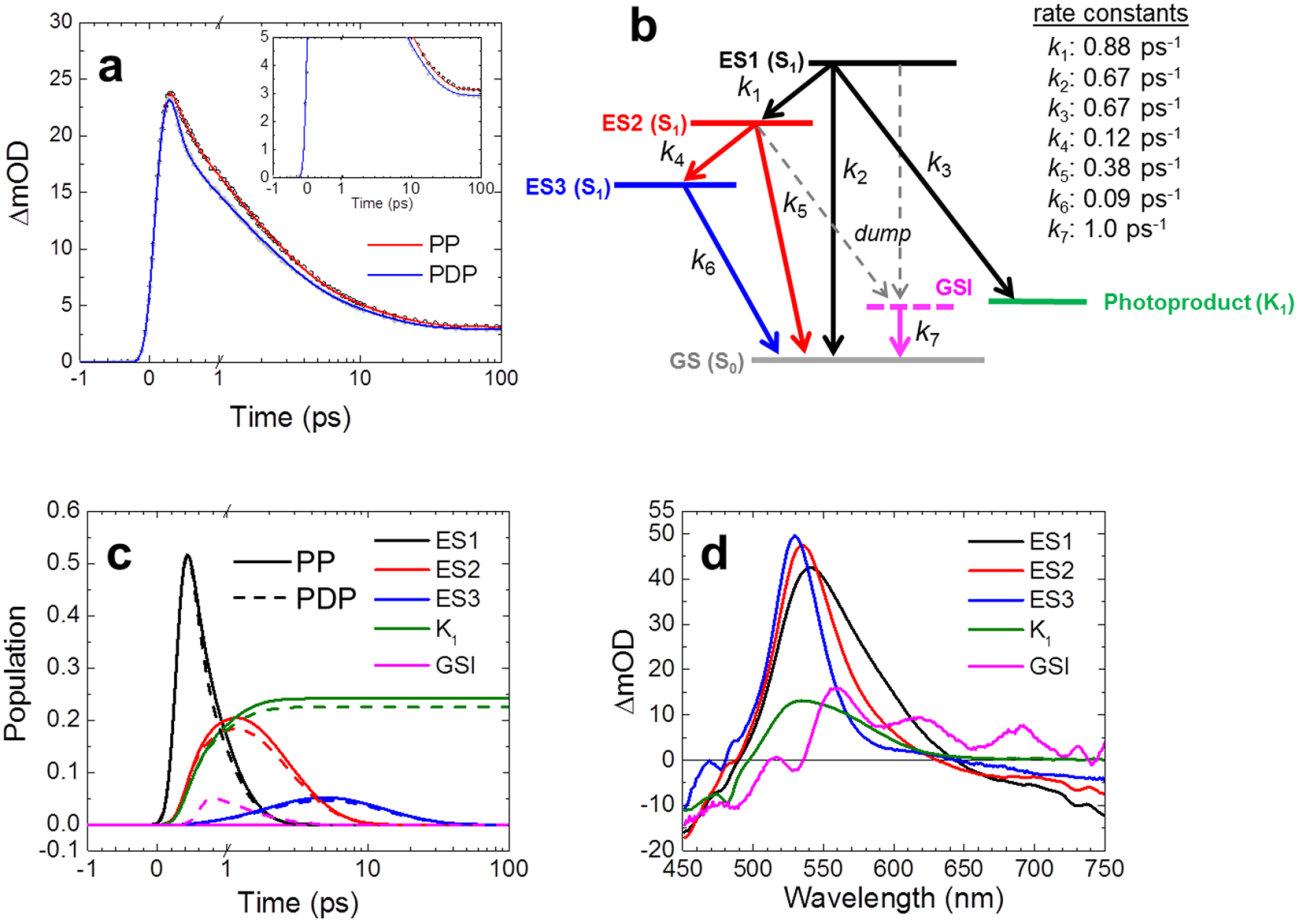
Pump-dump-probe data of C1C2 at pH 8 and target analysis results. (**a**), Time traces of pump probe (PP, open black dots with a red fitting line) and pump-dump-probe (PDP, closed gray dots with a blue fitting line) at 530 nm. In a and c, the time axis is linear until 1 ps, and logarithmic thereafter. (**b**), Kinetic scheme consisting of three excited states (ES1, ES2 and ES3), the first photoproduct state (K_1_) and ground state intermediate (GSI). The dump pulse at 300 fs converts a fraction of the excited states to the GSI. It affects ES1 and ES2, which is indicated by the dashed gray arrows. (**c**) Time evolution of populations of ES1, ES2, ES3, K_1_ and GSI. The solid and dashed lines represent pump-probe (PP) and pump-dump-probe (PDP) data, respectively. (**d**) Species-associated difference spectra (SADS) of pump-dump-probe experiments.

Fig. 3 shows the results of a pump-dump-probe experiment for C1C2 at pH 8, with pumping the ground state transition at 480 nm and dumping the SE at 720 nm at a time delay of 300 fs. Fig. 3a shows time traces of pump-probe and pump-dump-probe experiments probed at 530 nm, near the ESA and primary photoproduct absorption maxima (Figs S6 and S7 show raw and fitted pump-dump-probe results at 128 wavelengths at pH 8 and pD 8, respectively). An 11% signal decrease was seen in the pump-dump-probe time trace immediately after application of the dump pulse, which indicates that 11% of the excited molecules were forcibly relaxed the ground state by the dump pulse, and therefore do not participate in product formation any longer. At 100 ps (Fig. 3a, inset) where the excited states had completely decayed and only photoproduct absorption is observed, the photoproduct absorption was decreased only by 6.5% by the dump pulse. This experiment shows that a large fraction of product states have already been formed prior to application of the dump pulse. Given the early time of the dump (300 fs), it is likely that the photoproduct is preferentially formed by the 450 fs component. Indeed, we can quantitatively describe the pump-dump-probe data with product formation from the 450 fs component only (*vide infra*). This means that branching of the excited-state dynamics occurs and that a sequential model is not adequate.

### Primary photochemistry: target analysis

The femtosecond transient absorption and pump-dump-probe data on C1C2 show that during the first few picoseconds, a mixture of excited states and photoproducts make up the transient spectra. To disentangle the contributions by the various molecular species, we performed a target analysis wherein the data are described in terms of a kinetic scheme, thereby identifying their spectral signatures, estimate their lifetimes and their connectivity and quantum yield. In a target analysis, reactive compartments are associated in an explicit kinetic model, thus multiphasic decay and specific branching can be taken into account to obtain the difference spectra of the ‘pure’ molecular species involved in the reaction, so-called species-associated difference spectra (SADS)^34^.

To build the kinetic model, we need to consider the following aspects that become apparent from the transient absorption and pump-dump-probe experiments:

(i) We observe a progressive blue shifting and narrowing of the excited-state absorption on a sub-ps to ps timescale. Such phenomenology is indicative of vibrational relaxation processes in strongly vibronically coupled systems and/or evolution on the excited-state potential energy surface, as observed in protonated Schiff base retinal in solution^21^.
(ii) We observe three decay components of the stimulated emission of 450 fs, 2.0 ps and 11 ps. Such heterogeneity may follow from branched reaction dynamics in the excited state and from heterogeneity in the ground state. We will show below that both aspects may contribute.
(iii) The pump-dump-probe experiments indicate that the fast, 450 fs excited-state decay component produces the primary photoproduct, and that the slower components may not contribute.
(iv) To estimate the relative amplitude of the SADS (and thereby the photoproduct quantum yield), we assume that the ground-state bleach amplitude of the three excited states and of the photoproduct state should be roughly the same.
(v) Dumping the excited state in rhodopsins is expected to give rise to intermediates on the ground state potential energy surface^30^, which need to be included in the model.

The simplest adequate target model is shown in Fig. 3b. It includes three different excited states: ES1, ES2 and ES3 (on the S_1_ excited state potential energy surface), to reflect the observed three excited-state lifetimes. In addition, ES1, ES2 and ES3 represent the excited state at various levels of vibrational relaxation and evolution on the potential energy surface. ES1 is the initially populated shortest-lived excited state and branches to three other compartments: to ES2, the first photoproduct state K_1_ and the original ground state GS (S_0_). ES2 evolves to ES3 and GS, and ES3 evolves only to GS. A ground state intermediate (GSI) was populated only after application of a dump pulse, and evolves to GS. The target model was applied to transient absorption and pump-dump-probe datasets, of C1C2 dissolved in H_2_O and D_2_O, at pH/pD 8.

The target analysis results are shown in Fig. 3c,d (pH 8), and Fig. S5a,b (pD 8), respectively. At pH 8, ES1 branches to three other compartments at a total rate constant of 2.2 ps^−1^: to ES2 (40%), the first photoproduct state K_1_ (30%) and the original ground state GS (30%). ES2 is blue-shifted and narrowed with respect to ES1, indicating that this state has undergone vibrational relaxation. K_1_ is assigned as a well-known K-like state in microbial rhodopsins^35^. ES2 branches to ES3 and GS with rate constants of 0.12 and 0.38 ps^−1^, respectively. ES3 has progressively blue-shifted and narrowed with respect to ES2, indicating that this state has further undergone vibrational relaxation and/or structural relaxation on S_1_. ES3 evolved to GS with a rate constant of 0.09 ps^−1^. The GSI decayed to the GS with a rate constant of 1.0 ps^−1^. The GSI is characterized by a ground state bleach near 480 nm, and a broad absorption between 550 and 700 nm (Fig. 3d), indicating this species is red-shifted with respect to the steady-state ground state absorption of C1C2. Such GSI properties are similar to those observed previously in proteorhodopsin and PYP^26, 30^. It is uncertain whether the somewhat oscillatory spectral shape of the GSI is real or spurious. In the latter case it may follow from its low transient concentration in the spectral evolution and limits in data quality.

Here we consider the possibility that the dump pulse, which is resonant with the stimulated emission, also induces re-pumping of the excited state given that the ESA may extend to 720 nm. Repumping would result in the production of distinct photoproducts. However, the transient absorption and pump-dump-probe data can be adequately described by the kinetic model and the molecular compartments of Fig. 3b. The fit quality may be inspected in Figs S6 and S7 for 128 probed wavelengths. If any distinct photoproduct would have been formed due to repumping, such putative state should be visible in the spectral evolution. Such is clearly not the case, and we therefore conclude that no significant repumping of the excited state takes place. Target analysis results (SADS) for the transient absorption data at pH 8 are shown in Fig. S8c,d.

### Primary photochemistry: hybrid quantum mechanics/molecular mechanics calculations

**Figure 4:**
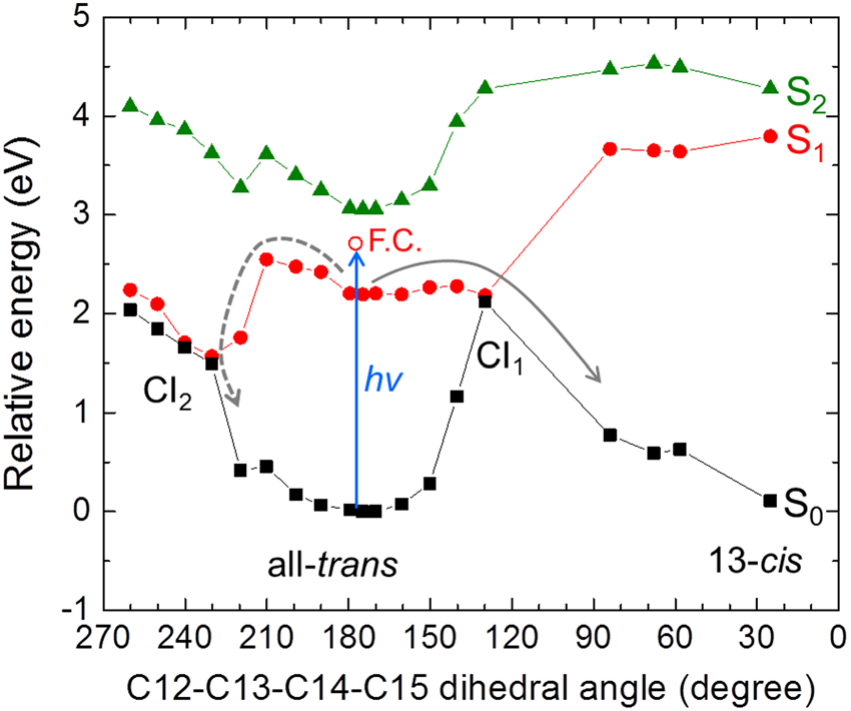
CASPT2//CASSCF(12,12) photoisomerization pathways from the all-*trans* retinal conformer, when the retinal Schiff base is hydrogen-bonded to Glu162. S_0_ (black closed squires with black solid line), S_1_ (red closed dots with red solid line) and S_2_ (green closed triangles with green solid line) energy profiles are shown. The blue arrow shows light excitation of all-*trans* retinal as performed on our pump-probe and pump-dump-probe spectroscopic experiments. The open red dot shows the Franck–Condon state (F.C.) The solid gray arrow shows the isomerization path through energy barrier of 1.96 kcal/mol (0.09 eV) on S_1_ via a conical intersection CI_1_. The dashed gray arrow indicates a relaxation path through energy barrier of 7.42 kcal/mol (0.32 eV) on S_1_ via another conical intersection CI_2_.

We performed CASPT2//CASSCF(12,12)/CHARMM calculations to study the excited-state photoisomerization of all-*trans* retinal in C1C2, in order to compare with the transient absorption, the pump-dump-probe results and the target analysis scheme. The calculations of C1C2 including a direct hydrogen bond between the retinal Schiff base and the side chain of Glu162, indicate a two-step retinal isomerization mechanism (Fig. 4): After S_0_ → S_1_^1^(π,π*) vertical excitation of all-*trans* retinal, the system relaxes from the Franck−Condon region to an S_1_ energy-minimum geometry (excess energy of 12.0 kcal/mol), mainly driven by partial inversion of the bond length alternation, as also evinced by S_1_ CASSCF/MM dynamics (see Fig. S9). Indeed, the S_0_ → S_1_ vertical excitation is optically much brighter than the eventual S_0_ → S_2_^1^(π,π*) excitation, as evinced by the difference in their transition oscillator strengths: 1.4 and 0.1, respectively. It follows a transition to the photoproduct state via a conical intersection (CI_1_). The C12–C13–C14–C15 dihedral angle of the retinal is *ca*. 180° on the ground state (S_0_) and S_1_ energy-minima, indicating the retinal isomer as all-*trans*. At the CI_1_, the dihedral angle evolved by a clockwise rotation to 130° (*i.e*. twisted by −50°) and finally relaxed to 24° (*i.e*. twisted by −156°), forming the 13-*cis* retinal isomer. Alternatively, CI_1_ can also lead back to the initial all-*trans* isomer (*i.e*. internal conversion). A different pathway was also found via another conical intersection (CI_2_), implying a counter-clockwise rotation after the initial vibrational relaxation to the S_1_ energy-minimum. Along the path, the C12–C13–C14–C15 dihedral angle evolves to 230° (*i.e*. twisted by 50°) at CI_2_, and relaxes back to 180° of the all-*trans* ground state. Starting from CI_2_, no ground state pathway leading to photoproduct formation was found. The energy barriers in the S_1_ state were calculated as 1.96 kcal/mol (0.09 eV) and 7.42 kcal/mol (0.32 eV) to reach CI_1_ and CI_2_, respectively. Those energy barriers are in agreement with previous CASPT2//CASSCF studies^36^.

**Figure 5:**
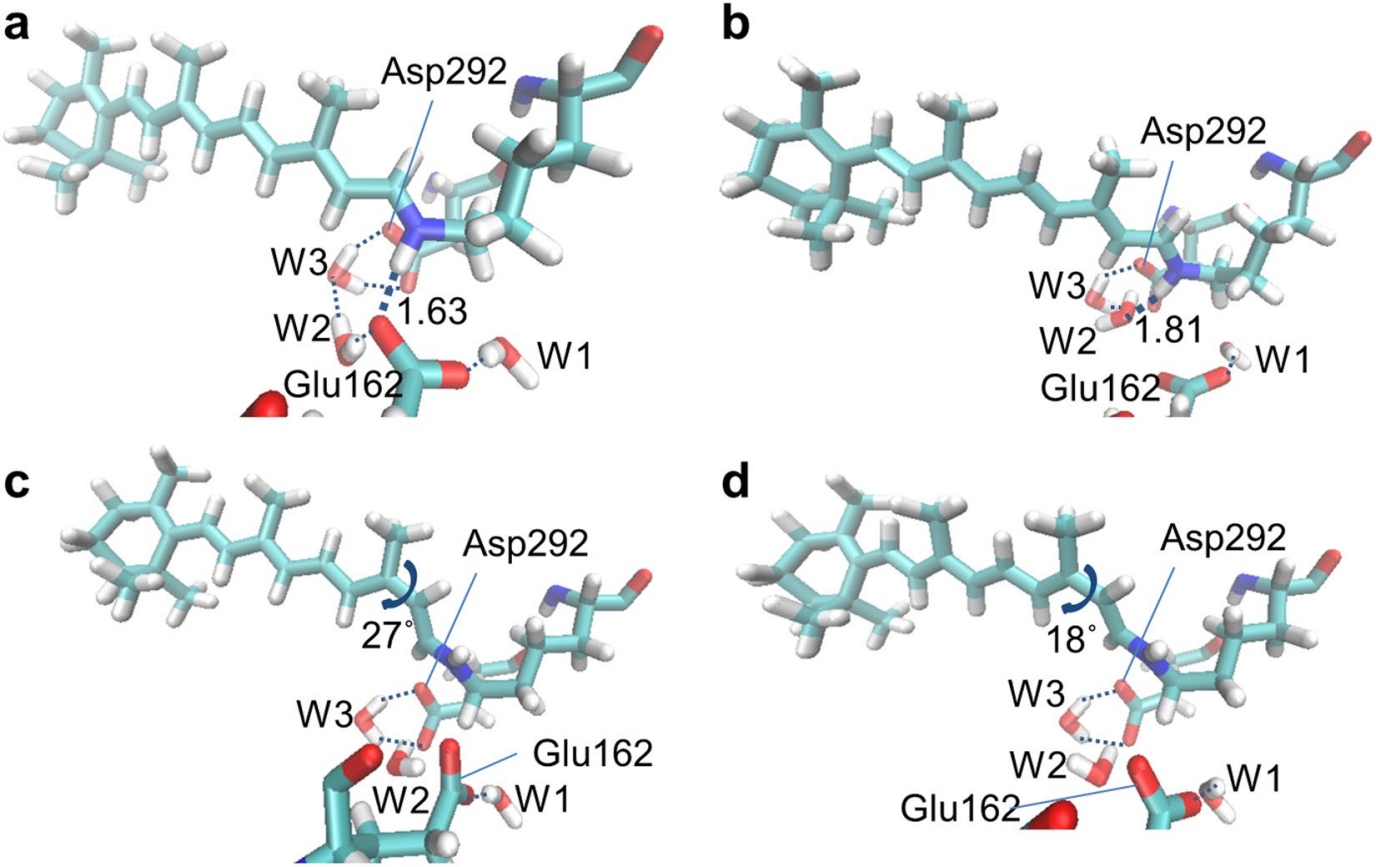
Calculated ground state minima structures. (**a,b**) all-*trans* and (**c,d**) 13-*cis* retinal-bound C1C2 active sites, including two possible hydrogen bonds to the retinal Schiff base for the all-*trans* conformations: to (**a**) Glu162 and to (**b**) a water molecule, W2.

The calculations predict heterogeneous excited-state dynamics because of the presence of the two barriers leading to CI_1_ and CI_2_, as we observed experimentally and in the target analysis. The productive path through CI_1_ can be assigned to the decay component of 450 fs, leading to the all-*trans* to 13-*cis* photoisomerization of the retinal and photoproduct K_1_. The unproductive path through CI_2_ likely involves the slower time components 2.0 ps and 11 ps, resulting in relaxation to the initial ground state. Nevertheless, the theoretical model predicts a single energy barrier to reach CI_2_ (see Fig. 4). This was also found in a previous work using a similar computational approach to C1C2^36^. Hence, in the context of the theoretical model, to explain the presence of three different time components we postulate a variety of ground state active site retinal pockets, as was depicted in a recent study of ChR2^19^. Indeed, Guo *et al*. propose three hydrogen-bonded networks, involving a direct hydrogen bond to the Glu side chain, to the Asp side chain or to a water molecule. In C1C2, a hydrogen bond with Glu162, not with Asp292, was experimentally demonstrated^18^. Hence, we propose as alternative network a hydrogen bond between retinal and water. The differences between both active sites can be seen in Fig. 5, where both optimized all-*trans* and 13-*cis* conformations are shown: a water molecule (W2) can compete with Glu162 in the formation of the hydrogen bond with the retinal Schiff base. This alternative active site conformation is indeed related to different excited state energy barriers to reach CI_2_, as recently discussed also in the study of a retinal-like biomimetic photoswitch^37^. In any case, as demonstrated by the single time constant involved in the photoproduct formation through CI_1_, this ground state heterogeneity does not affect the clockwise rotation of the retinal in the excited state (see Fig. S10): the energy barriers in the S_1_ state were calculated as 2.10 kcal/mol (0.09 eV) to reach CI_1_ − *i.e*. almost the same as in Fig. 4− and 5.20 kcal/mol (0.23 eV) to reach CI_2_ − *i.e*. 2.2 kcal/mol lower than in Fig. 4−, respectively, remaining almost unaltered the S_1_ excess energy from the Franck−Condon to minimum region (12.2 kcal/mol) and the S_0_ → S_1,2_ oscillator strengths. Thus, we conclude that the isomerization via CI_1_ proceeds in 450 fs regardless of the hydrogen-bond network on the ground state, while the relaxation to the initial ground state via CI_2_ occurs in 2.0 ps or 11 ps, depending on the ground-state hydrogen-bond network.

These conclusions highlight that, in spite of structural similarities between different rhodopsins, both electronic ground- and excited-states can result in different scenarios. Especially, rhodopsin (Rh) and bathorhodopsin (batho-Rh) have shown a single ground state conformation and a single excited state pathway, leading to the S_1_/S_0_ conical intersection in around 100 fs, hence without finding S_1_ minima^38, 39^. On the other hand, C1C2 is definitely a more complex system, where ground state heterogeneity and excited state minima should be considered for interpreting the experimental data.

The kinetic model of Fig. 3b assumes that the multiexponential excited-state decay arises exclusively from branching dynamics in the excited state. However, as argued above, dark state heterogeneity is likely present which will manifest itself as a biexponential, parallel excited-state decay that would be mixed in with the excited-state relaxation process, complicating the kinetic analysis. However, under the assumption that the spectral shape of the excited-state intermediates and the vibrational relaxation dynamics does not vary appreciably between the two dark-state conformers, the model of Fig. 3b is equivalent to such extended parallel model.

### Quantum yield of primary photoproduct K_1_ formation

When comparing the ground state bleach amplitudes in the EADS of Fig. 2, the 630 ps EADS has an amplitude of about 25% of that of the 450 fs component, which indicates that about 25% of the initially excited molecules end up in the primary photoproduct K_1_ (we refer to Fig. S3 for the original traces). However, estimations on the basis of such simple considerations may be problematic because of partly compensating bleach and absorption features in the transient absorption spectra. For a more systematic approach that takes into account the rich spectral and temporal evolution of the system, we applied the target analysis of Fig. 3, where the obtained SADS represent pure molecular states and hence have to conform to realistic spectral shapes. Here, we set the additional condition that the ground state bleach amplitudes should be similar. The kinetic modelling resulted in a quantum yield of 30 ± 3%, which is similar to that estimated above from the sequential analysis.

**Figure 6:**
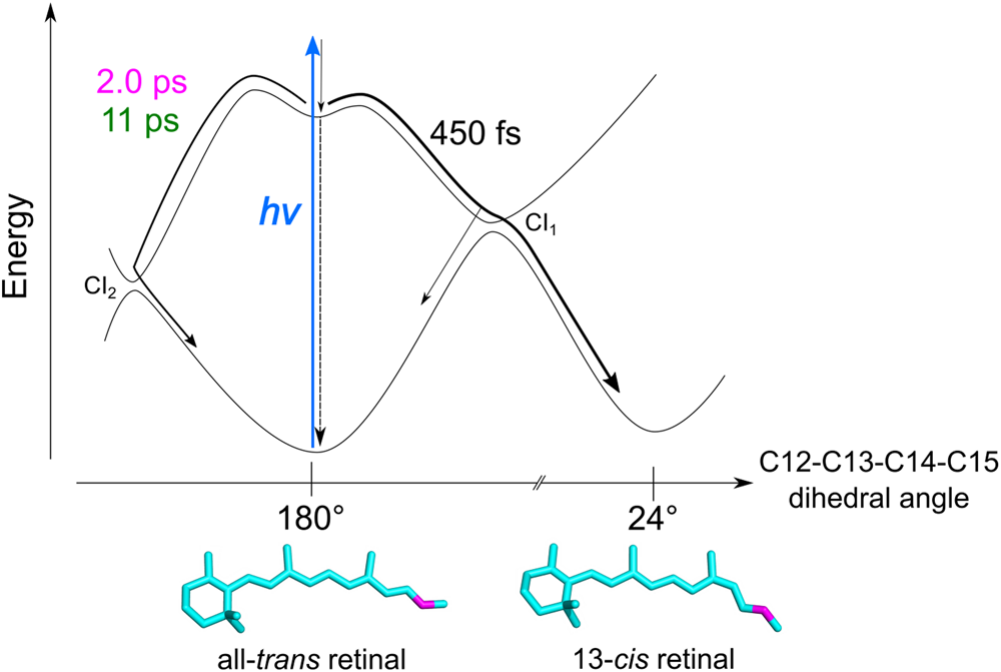
Energy diagram on excited-state dynamics of C1C2. After photon absorption, two distinct paths are suggested on the excited-state dynamics: isomerization path via CI_1_ with 450 fs and relaxation path to the initial ground state via CI_2_ with 2.0 ps and 11 ps.

The quantum yield of K_1_ state formation as estimated from the target analysis, 30%, is low as compared to bacteriorhodopsin, where it is around 60%^40^. According to the target model, the losses have two causes: (i) the presence of nonproductive fractions in excited state decay, *i.e*. the 2.0 and 11 ps components which make up 40% of the excited-state decay, and (ii) a nonunity quantum yield of the 450 fs component. The 450 fs component constitutes 60% of excited-state decay, which means that half of those excited states proceed to the photoproduct K_1_, and the other half relaxes to the original ground state through CI_1_. Thus, the 450 fs component inherently has a 50% quantum yield, which is in line with bacteriorhodopsin that follows a single path on the potential energy surface. This is another indication that the 450 fs component does not follow the CI_2_ reaction path. The schematic excited-state reaction diagram is shown in Fig. 6.

### Isomeric composition of C1C2

A key question concerns the isomeric composition of C1C2 in the dark and in our experimental conditions. In the dark, C1C2 assumes an all-*trans* retinal chromophore, as evidenced from solid-state NMR^41^ and the X-ray structure^13^. ChR2 also adopts an all-*trans* retinal chromophore in the dark^42^. There is evidence from resonant Raman spectroscopy that in ChR2/H134R, moderate illumination leads to a minority population of a 13-*cis*/15-*syn* conformer^41^, but such has not been systematically investigated for C1C2. Fig. S11a,b shows the C1C2 absorption spectra and their 2^nd^ derivatives of a dark-adapted sample and the same sample after two hours of 0.2 mW laser illumination in the Lissajous scanner, which is the same illumination condition as our transient absorption experiments. The spectra are virtually identical, with the same absorption maxima before and after illumination. Fig. S11c shows the stimulated Raman spectrum of the C1C2 dark state, with preresonant Raman pumping at 800 nm. It shows a C=C stretch band near 1560 cm^−1^ and a C=N stretch at 1660 cm^−1^, which are indicative of an all-*trans* chromophore^41, 43^. From these data, we conclude that under our experimental circumstances, C1C2 retains an all-*trans* retinal chromophore.

### Comparison of the ultrafast reaction dynamics with other channelrhodopsins

Here we compare the excited-state dynamics of C1C2 with other channelrhodopsins: ChR2 from *Chlamydomonas reinhardtii* ^15–17^ and *Ca*ChR1^44–46^, which is a channelrhodopsin found in *Chlamydomonas augustae* ^47^. By visible pump-probe^15, 16^, visible-pump/IR-probe^17^ and upconversion^15^ experiments, it was concluded that the formation of the first photoproduct state occurs in ~400 fs in ChR2. As discussed above, the time constant of the retinal isomerization in C1C2 is 450 fs, which is close to that in ChR2. However, a clear difference was seen in the excited-state relaxation mechanisms to the initial ground state. In ChR2, the stimulated emission decays almost completely with ~400 fs^15, 16^, indicating relaxation to the ground state in this time frame. On the other hand, in C1C2, three components of 450 fs, 2.0 ps and 11 ps, of which the latter two make up 40% of the excited-state decay, are related to the relaxation to the ground state. Because five helices (TM1–TM5) among the seven transmembrane helices are originated from ChR1, the conformation of important residues for retinal dynamics such as Glu162 may be significantly different between C1C2 and ChR2 and should be similar to ChR1. Possibly, the conformational difference of residues such as Glu162 affects the relaxation process of the excited molecules. Further investigation *e.g*. with time-resolved vibrational spectroscopy in C1C2 would be helpful for deeper understanding of the relaxation process. In *Ca*ChR1, it was found that isomerization occurs much faster than ChR2 and C1C2, in 110 fs^44–46^. In that sense, C1C2 behaves more similar to ChR2 than *Ca*ChR1. However, *Ca*ChR1 has a red-shifted absorption (at ~520 nm) and different photocurrent kinetics from *Chlamydomonas reinhardtii* ChR1^47^, thus the *Ca*ChR1 photoinduced kinetics may be significantly different from that of *Chlamydomonas reinhardtii* ChR1 and ChR2.

### C1C2 pump-dump-probe spectroscopy: consequences for superresolution optogenetics

We have demonstrated that it is possible to dump the excited state of C1C2. This finding suggests that it might be possible to establish superresolution optogenetics based on channelrhodopsin by means of STED/RESOLFT techniques: in such a case, a combination of blue activation and near-IR deactivation beams would be used to activate neurons with sub-diffraction resolution, particularly when optogenetics at the organelle level is considered^48^. However, the properties that we have determined for the excited-state reaction dynamics of C1C2 are not yet favorable for such applications. The excited-state lifetime is multiphasic, with lifetimes of 450 fs, 2.0 ps and 11 ps. We have shown that only the first, fast phase of 450 fs activates C1C2, which means that during this short time the excited states need to be dumped in a STED-like configuration. We find that dumping even at very early delays (300 fs) results in only partial effects on product formation (*i.e*. an 11% dumping of excited states leads to only 6.5% loss of photoproduct). For superresolution purposes, the proteins in the deactivation beam need to be deactivated by almost 100%, which is nearly impossible with the C1C2 short reactive lifetimes.

### Slow photoproduct dynamics of C1C2

Next, we discuss the photoproduct dynamics after the K_1_ state formation in C1C2. As discussed above, 450 fs, 2.0 ps and 11 ps lifetimes are involved in the excited-state dynamics, indicating that the slower components are related to the photoproduct ground state dynamics of C1C2 at pH 8 (Fig. 2a, bottom panel) and pH 10 (Fig. 2b, bottom panel). At pH 8, the photoproduct absorption shows a red shift from 523 nm to 530 nm in 630 ps (Fig. 2a, green to magenta evolution), which can be assigned as a second K-like state formation as proposed for ChR2^16^, hereafter referred to as K_2_. A similar red shift was seen at pH 10 (in 650 ps, Fig. 2b). In the 490 ns evolution (Fig. 2a, magenta to cyan evolution), a ~440-nm signal rises and the ~530-nm signal decreases to a lower amplitude. The ~440-nm signal rise can be assigned to formation of an L-like state, which is a blue-shifted intermediate widely seen in microbial rhodopsins^35, 49^. In bacteriorhodopsin, it was suggested that the retinal Schiff base is located closer to the counterions in the L state than in the K state. As seen in Fig. 2a, the 530-nm absorption remains with the increase of the 440-nm absorption in 490 ns, implying the L-like state exists in equilibrium with the K-like state in C1C2 at pH 8.

**Figure 7:**
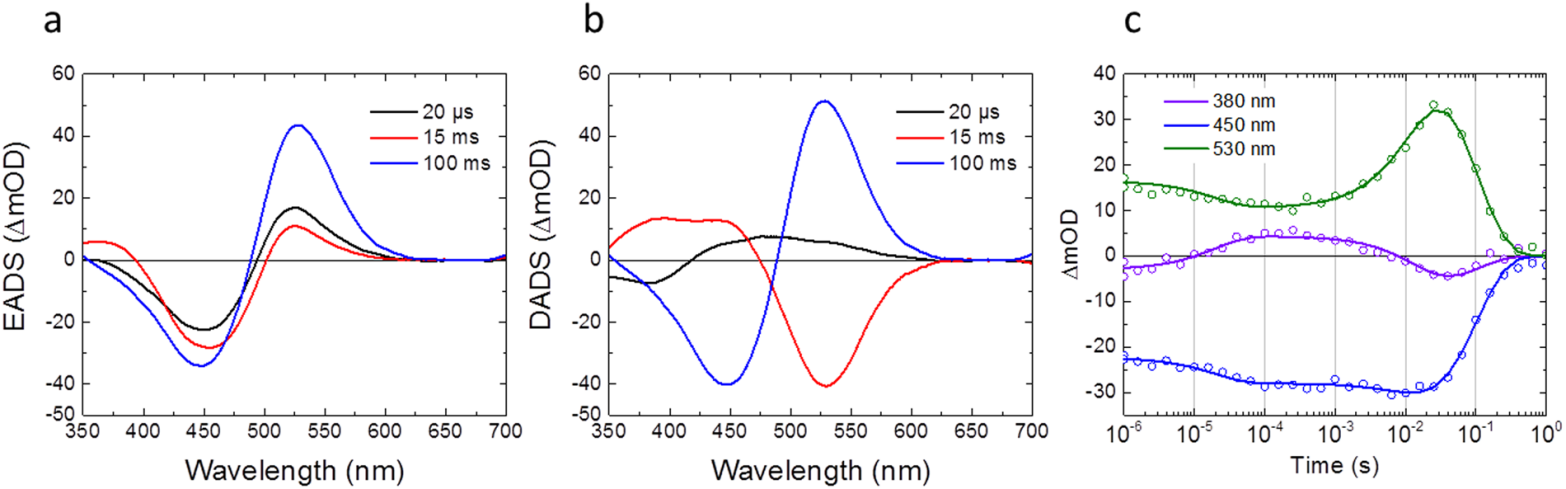
Globally fitted spectra and selected time traces of flash photolysis experiments in C1C2 at pH 8. **(a)** EADS, **(b)** DADS and **(c)** time traces with fitting curves (solid lines) at 380 (purple), 450 (blue) and 530 nm (green).

Furthermore, we performed flash photolysis experiments to investigate slow photoproduct dynamics from 1 µs to 10 s in C1C2 at pH 8 and 10 (Fig. S12). EADS, DADS and time traces at 380 nm, 450 nm and 530 nm at pH 8 and pH 10 are shown in Fig. 7 and Fig. S13, respectively. In Fig. 7a, three exponential components were seen for C1C2 at pH 8: 20 µs, 15 ms and 100 ms. In 20 µs, a near-UV absorption at <400 nm appeared, which is assigned to the M intermediate that has a deprotonated retinal. Notably, a decay of a broad peak at 430–600 nm was observed in 20 µs (Fig. 7b), which are probably derived from a mixture of the K and L intermediates, while a significant amount of the K state absorption (~530 nm) remained (Fig. 7a). Furthermore, an L-intermediate decay (at ~440 nm) is also seen in the 15-ms DADS component (Fig. 7b), which implies the L intermediate partially remains after 20 µs. These observations indicate that K/L/M equilibrium is formed after 20 µs, similarly to sodium ion pump rhodopsin KR2^50, 51^. Significantly, the amplitude of the M state signal of C1C2 at pH 8 is ~3-fold less than that of ChR2 at pH 7.4^15^, indicating that in C1C2 the K/L/M equilibrium is tilted towards K and L. In the pump-probe spectra shown in Fig. 2a, the M state absorption was not clearly visible and the ~20 µs component was not detected, which relate to noise issues in the near-UV that result from the decreased intensity of the white light continuum in this spectral region.

**Figure 8:**
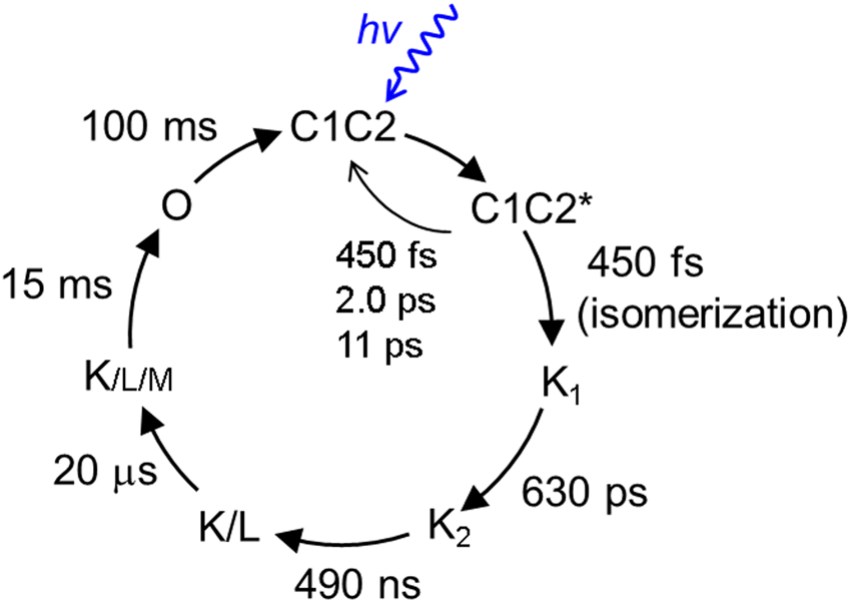
Photocycle models of C1C2 at pH 8.

In 15 ms, the L (~440 nm) and M (<400 nm) intermediates decayed, while another 530-nm absorbing component appeared, which is assigned as the ion-conducting O intermediate that has a reprotonated retinal. In 100 ms, the O state absorption disappeared with recovery of the GSB signal, which indicates the photocycle is completed in 100 ms. The photocycle model of C1C2 at pH 8 is shown in Fig. 8.

C1C2 at pH 10 has a similar photocycle as that at pH 8, with the key difference that the transient M concentration is higher, indicating that the K/L/M equilibrium is more tilted towards M (Fig. S14). Judging from the amplitudes of the M and O intermediates, it shows almost the same M-intermediate population as ChR2 at pH 7.4^15^ (Fig. S13). Possibly, pKa values of important residues for the proton transfer (e.g. counterions of the retinal Schiff base) are between 8 and 10, thus affecting the deprotonation of the retinal Schiff base and transient equilibria between its protonated and deprotonated forms.

## Conclusion

We investigated the excited-state and photoproduct-state dynamics of a functional chimeric channelrhodopsin C1C2, with known X-ray structure and which has properties similar to *Chlamydomonas reinhardtii* ChR1 and ChR2 with respect to absorbance, ion selectivity and photocurrent kinetics. With transient absorption and pump-dump-probe spectroscopy together with target analysis and QM/MM calculations, we demonstrated that the retinal isomerization in C1C2 occurs in 450 fs after photon absorption with an estimated quantum yield of 30% via a conical intersection CI_1_ that is reached after clockwise rotation of the C13=C14 bond. Relaxation to the initial ground state, which is a competing process of the isomerization, proceeds with three time components: 450 fs (via CI_1_), 2.0 ps and 11 ps via a second conical intersection CI_2_ that is reached after counterclockwise rotation of the C13=C14 bond. The presence of the additional conical intersection CI_2_ lies at the basis of the low (30%) isomerization quantum yield determined in this study. Structural heterogeneity on the ground-state hydrogen-bond network around all-*trans* retinal possibly contributes to the distinct relaxation with 2.0 ps and 11 ps, but does not affect the isomerization with 450 fs. Furthermore, femtosecond to second photocycle of C1C2 has been determined. Strikingly, at pH 8 such M-like state is seen less significantly in C1C2 than in ChR2, while the C1C2 protein shows higher ion conducting. Thus, clear differences were observed in amplitude of the M-like state rise between C1C2 and ChR2, which indicates that the proton transfer mechanism and/or the transient equilibria between protonated and deprotonated forms of the retinal Schiff base is different in C1C2 and ChR2. Our findings can be considered a crucial step toward the elucidation of channelrhodopsin photoactivation.

## Methods

### Sample preparation

The C1C2 chimera (1–348 amino acids) was expressed and purified from *P. pastoris* according to previously described protocol^41^. Protein was concentrated to 520 μM in 20 mM Tris-HCl containing 150 mM NaCl and 0.03% β-D-maltoside (DDM) at pH 8.0. Buffers were exchanged by repeated dilution-concentration steps (Amicon Ultra 100000 MWCO, Millipore) to pH 10.0 (20 mM CAPS buffer with 150 mM NaCl) containing 0.03% DDM. Protein samples were placed between two CaF_2_ plates separated by a 400 μm sample spacer. The sample holder was set on the Lissajous scanner as reported previously^43^ to excite a fresh part of the samples in each pump-probe and pump-dump-probe data acquisition process^52^.

### Transient absorption spectroscopy

Femtosecond to sub-millisecond transient absorption measurements were performed by a pump-probe setup with synchronized 1 kHz Ti:Sapphire amplifiers as reported previously^53–55^. A CaF_2_ plate was used for supercontinuum white light generation, and selected wavelength regions; 380–680 nm, were detected by the photodiode array. The time delay was varied up to 125 μs at 169 data points with the minimum temporal step of 50 fs. The diameters of the pump and the probe beams at the sample position were ~200 μm and ~70 μm, respectively. The wavelength of the pump beam was centered at 480 nm, and attenuated to ~400 nJ. The instrument response function was ~150 fs, estimated from global fitting. The flash photolysis experiments were performed as reported previously to observe transient absorption from 1 µs to 10 s^43^.

### Pump-dump-probe spectroscopy

Multi-pulse visible pump-dump-probe measurements were performed on a setup reported previously^29, 31^. The central wavelength of the pump and dump pulses were set to 480 nm (~0.8 μJ/pulse) and 720 nm (~1.5 μJ/pulse), respectively. The pump and dump pulses were modulated using choppers to 500 Hz and 250 Hz, respectively. On the probe line, an 800-nm pulse was focused on a 2-mm sapphire plate for supercontinuum white light generation. Two optical stages (IMS-6000, Newport) were installed on the pump and dump lines, and the temporal difference between the pump and dump pulses was fixed as 300 fs. The time delay between the pump and probe was covered from −50 ps to +100 ps with the shortest step of 20 fs (Fig. S1). The pump-dump-probe spectra were globally fitted, and the instrumental response function was ~240 fs, estimated from the global fitting.

### Global analysis and target analysis

The data were fitted by using global and target analysis^34^, with the extension for pump-dump-probe data described previously^25, 32^. In global analysis, all wavelengths are analyzed simultaneously and a set of common time constants and spectra is produced^34^. In a sequential analysis (1 → 2 → 3 → ….) the numbers indicate evolution-associated difference spectra (EADS) that interconvert with successive mono-exponential decay times, each of which can be regarded as the lifetime of each EADS. The first EADS corresponds to the time-zero difference spectrum. The first EADS evolves into the second EADS with time constant τ_1_, which in turn evolves in the third EADS with time constant τ_2_, etc. This procedure clearly visualizes the evolution of the excited and intermediate states of the system. In general, the EADS may well reflect mixtures of difference spectra of pure electronic states, which may arise from heterogeneous ground states or branching at any point in the photo-induced evolution^56–58^. It is important to note that sequential analysis is mathematically equivalent to a parallel, sum-of-exponential analysis, which produces decay-associated difference spectra (DADS). The analysis program calculates both EADS and DADS and the time constants that follow from the analysis apply to both^20^. For a more detailed description of global analysis we refer to Van Stokkum *et al*. 2004^34^. The standard errors in the time constants were 10%^56, 59^.

### Computational methods

The monomer of C1C2 was built by using the crystal structure 3UG9 as template^13^, where the retinal shows an all-*trans* conformation. A hybrid quantum mechanics/molecular mechanics (QM/MM) model was set, by including the retinal chromophore in the QM region. More in detail, considering its successful results in describing quantitatively the energies and electronic structure of rhodopsin proteins^36, 38, 39^ and retinal-based molecules^60–62^, the MS-CASPT2//SA-CASSCF methodology^63^ was applied, including the whole π system of the retinal in the active space (*i.e*. 12 electrons in 12 molecular orbitals). More in detail, the State Average-Complete Active Space Self Consistent Field (SA-CASSCF) method was applied to optimize the system, followed by MS-CASPT2 (Multi State-Complete Active Space Perturbation Theory to Second Order) energy calculation on top of the SA-CASSCF optimized geometries, performed to include the dynamic electron correlation by second-order perturbation theory. Indeed, the CASSCF method includes only the static electron correlation, hence allowing only a qualitative description of the system, while the inclusion of the dynamic electron correlation permits to reach a quantitative description of the ground and excited state energies.

CASSCF optimizations and CASPT2 single-point energy corrections were performed using 3 roots, *i.e*. the ground state (S_0_) and the two lowest-lying singlet excited states (S_1_ and S_2_). Indeed, even if we are interested in the evolution of S_1_ reaching S_0_ through conical intersections, also S_2_ was included for a better description of the system electronic structure. Moreover, CASPT2 calculations were performed using an imaginary shift of 0.2, as conventionally applied to avoid eventual problems of intruder states. The IPEA shift was set to 0.0.

The CHARMM22 classical force field^64^ was applied to the rest of the protein, while the TIP3P model^65^ was used to describe water molecules. Considering the high resolution of the X-ray data (2.3 Å)^13^ and the aim to model the ultrafast photoinduced events after light absorption, all atom positions were kept to their crystal structure values, letting relax the active site, composed by the retinal and its nearby pocket (see below).

In order to correctly treat the frontier between the QM and the MM region, a hydrogen link atom was placed between the nitrogen atom of the Schiff base and the first carbon atom of the lysine residue, Cε. This allowed to include the whole chromophore in the QM region while keeping a reduced amount of atoms to be treated by the computationally expensive multiconfigurational quantum chemistry method MS-CASPT2//SA-CASSCF. During QM/MM optimizations, the Morokuma scheme was used, *i.e*. the hydrogen link atoms was always kept along the line connecting the frontiers’ QM and MM atoms.

The QM/MM (MS-CASPT2//SA-CASSCF/CHARMM) model applied ensures electrostatic and mechanical embedding through the Molcas/Tinker interface, *i.e*. the QM region is polarized by the MM point charges around. In order to avoid overpolarization of the QM wavefunction due to the presence of the hydrogen link atom, the charge of the Cε atom and of the two connected hydrogens was set to zero and redistributed on the rest of the atoms composing the lysine and the two neighboring amino acids.

During the QM/MM optimization, all atoms of the side chains and water molecules around the retinal – forming the retinal binding pocket – were allowed to move, following the micro-iterations scheme, that is converging the MM subsystem geometry every QM step. More in detail, the following residues were included: side chains of the lysine covalently bound to the retinal, Glu162, Trp163, Thr166, Cys167, Trp262, Ser295, Pro266, Trp299, Met294, Ile170, Cys298, Val125, Glu129, Asp292, Thr198, Leu221, Phe269, Phe217 and 6 water molecules. The rest of the protein and water molecules were kept at their crystallographic position, in order to keep the overall tertiary structure of the monomer. Indeed, the modeled retinal isomerization takes place in few hundreds of fs, a time which does not allow a full protein (including backbone) relaxation.

Both, all-*trans* and 13-*cis* retinal-bound C1C2, were optimized on the ground state, followed by a calculation of the S_1_ minimum energy path from the all-*trans* Franck−Condon region to the S_1_ minimum. Subsequently, it was performed an all-*trans*-to-13-*cis* relaxed scan on the optically bright state (S_1_), along the photoisomerizable dihedral angle. The 6–31 G(d) basis set was adopted for all optimizations, followed by energy calculations with the ANO-L-VDZP basis set. Different hydrogen-bonding patterns to the retinal Schiff base were considered, by CASSCF/MM optimization on the ground state: hydrogen bond (*i*) to deprotonated Glu162, as experimentally observed^18^ and (*ii*) to a water molecule, as suggested computationally^66^. In the case (*i*) a S_1_ CASSCF/MM excited state dynamics was additionally performed, considering the optimized ground state structure as initial geometry and without initial kinetic energy (*i.e*. temperature = 0 K). A time step of 1 fs was adopted for integration of the Velocity Verlet algorithm, keeping the total energy constant along the dynamics. This trajectory has the scope of giving more insights into the initial vibrational relaxation process upon light absorption, *i.e*. from the Franck−Condon to the S_1_ minimum region. All calculations were performed with Molcas 7.8^67^ interfaced to Tinker 5^68^.

### Stimulated Raman spectroscopy

Steady-state Raman spectrum of the dark-adapted state of C1C2 at pH 8 was obtained by watermarked stimulated Raman spectroscopy as previously reported^51, 69^. Raman pump (800 nm, ~15 μJ) and Raman probe (~840–960 nm) were spatiotemporally overlapped at the sample position with the diameter of ~100 μm.

## Electronic supplementary material

supplementary information

## References

[1] Nagel G (2002). Channelrhodopsin-1: a light-gated proton channel in green algae. Science.

[2] Nagel G (2003). Channelrhodopsin-2, a directly light-gated cation-selective membrane channel. Proceedings of the National Academy of Sciences of the United States of America.

[3] Boyden ES, Zhang F, Bamberg E, Nagel G, Deisseroth K (2005). Millisecond-timescale, genetically targeted optical control of neural activity. Nature neuroscience.

[4] Hegemann P, Moglich A (2011). Channelrhodopsin engineering and exploration of new optogenetic tools. Nature methods.

[5] Deisseroth K (2011). Optogenetics. Nature methods.

[6] Fenno L, Yizhar O, Deisseroth K (2011). The development and application of optogenetics. Annual review of neuroscience.

[7] Prigge M (2012). Color-tuned channelrhodopsins for multiwavelength optogenetics. The Journal of biological chemistry.

[8] Lin JY, Knutsen PM, Muller A, Kleinfeld D, Tsien RY (2013). ReaChR: a red-shifted variant of channelrhodopsin enables deep transcranial optogenetic excitation. Nature neuroscience.

[9] Kleinlogel S (2011). Ultra light-sensitive and fast neuronal activation with the Ca(2)+ -permeable channelrhodopsin CatCh. Nature neuroscience.

[10] Wietek J (2014). Conversion of channelrhodopsin into a light-gated chloride channel. Science.

[11] Berndt A, Lee SY, Ramakrishnan C, Deisseroth K (2014). Structure-guided transformation of channelrhodopsin into a light-activated chloride channel. Science.

[12] Dawydow A (2014). Channelrhodopsin-2-XXL, a powerful optogenetic tool for low-light applications. Proceedings of the National Academy of Sciences of the United States of America.

[13] Kato HE (2012). Crystal structure of the channelrhodopsin light-gated cation channel. Nature.

[14] Kato HE (2015). Atomistic design of microbial opsin-based blue-shifted optogenetics tools. Nature communications.

[15] Verhoefen MK (2010). The photocycle of channelrhodopsin-2: ultrafast reaction dynamics and subsequent reaction steps. Chemphyschem: a European journal of chemical physics and physical chemistry.

[16] Scholz F, Bamberg E, Bamann C, Wachtveitl J (2012). Tuning the primary reaction of channelrhodopsin-2 by imidazole, pH, and site-specific mutations. Biophysical journal.

[17] Neumann-Verhoefen MK (2013). Ultrafast infrared spectroscopy on channelrhodopsin-2 reveals efficient energy transfer from the retinal chromophore to the protein. Journal of the American Chemical Society.

[18] Ito S (2014). Water-containing hydrogen-bonding network in the active center of channelrhodopsin. Journal of the American Chemical Society.

[19] Guo Y (2016). Active Site Structure and Absorption Spectrum of Channelrhodopsin-2 Wild-Type and C128T Mutant. Chem. Sci..

[20] Toh KC, Stojkovic EA, van Stokkum IH, Moffat K, Kennis JT (2011). Fluorescence quantum yield and photochemistry of bacteriophytochrome constructs. Phys. Chem. Chem. Phys..

[21] Hamm P (1996). Femtosecond spectroscopy of the photoisomerisation of the protonated Schiff base of all-trans retinal. Chemical Physics Letters.

[22] Mathies RA, Brito Cruz CH, Pollard WT, Shank CV (1988). Direct observation of the femtosecond excited-state cis-trans isomerization in bacteriorhodopsin. Science.

[23] Gai F, McDonald JC, Anfinrud PA (1997). Pump-dump-probe spectroscopy of bacteriorhodosin: Evidence for a near-IR excited state absorbance. Journal of the American Chemical Society.

[24] Ruhman S, Hou BX, Friedman N, Ottolenghi M, Sheves M (2002). Following evolution of bacteriorhodopsin in its reactive excited state via stimulated emission pumping. Journal of the American Chemical Society.

[25] Kennis JTM (2004). Uncovering the hidden ground state of green fluorescent protein. Proceedings of the National Academy of Sciences of the United States of America.

[26] Larsen DS (2004). Incoherent manipulation of the photoactive yellow protein photocycle with dispersed pump-dump-probe spectroscopy. Biophysical journal.

[27] Kim PW (2012). Second-Chance Forward Isomerization Dynamics of the Red/Green Cyanobacteriochrome NpR6012g4 from Nostoc punctiforme. Journal of the American Chemical Society.

[28] Kim PW (2013). Unraveling the Primary Isomerization Dynamics in Cyanobacterial Phytochrome Cph1 with Multipulse Manipulations. Journal of Physical Chemistry Letters.

[29] Kennis JTM, van Stokkum IHM, Peterson DS, Pandit A, Wachter RM (2013). Ultrafast Proton Shuttling in Psammocora Cyan Fluorescent Protein. Journal of Physical Chemistry B.

[30] Rupenyan A (2009). Reaction Pathways of Photoexcited Retinal in Proteorhodopsin Studied by Pump-Dump-Probe Spectroscopy. Journal of Physical Chemistry B.

[31] Di Donato M (2011). Proton transfer events in GFP. Physical Chemistry Chemical Physics.

[32] van Oort B, ter Veer MJT, Groot ML, van Stokkum IHM (2012). Excited state proton transfer in strongly enhanced GFP (sGFP2). Physical Chemistry Chemical Physics.

[33] van Oort B, van Grondelle R, van Stokkum IHM (2015). A Hidden State in Light-Harvesting Complex II Revealed By Multipulse Spectroscopy. Journal of Physical Chemistry B.

[34] van Stokkum IH, Larsen DS, van Grondelle R (2004). Global and target analysis of time-resolved spectra. Biochimica et biophysica acta.

[35] Ernst OP (2014). Microbial and animal rhodopsins: structures, functions, and molecular mechanisms. Chem Rev.

[36] Dokukina I, Weingart O (2015). Spectral properties and isomerisation path of retinal in C1C2 channelrhodopsin. Physical chemistry chemical physics: PCCP.

[37] Garcia-Iriepa C (2016). A biomimetic molecular switch at work: coupling photoisomerization dynamics to peptide structural rearrangement. Physical chemistry chemical physics: PCCP.

[38] Polli D (2010). Conical intersection dynamics of the primary photoisomerization event in vision. Nature.

[39] Schapiro I (2011). The ultrafast photoisomerizations of rhodopsin and bathorhodopsin are modulated by bond length alternation and HOOP driven electronic effects. Journal of the American Chemical Society.

[40] Tittor JaOD (1990). The quantum yield of bacteriorhodopsin. FEBS letters.

[41] Bruun S (2015). Light-Dark Adaptation of Channelrhodopsin Involves Photoconversion between the all-trans and 13-cis Retinal Isomers. Biochemistry.

[42] Becker-Baldus J (2015). Enlightening the photoactive site of channelrhodopsin-2 by DNP-enhanced solid-state NMR spectroscopy. Proceedings of the National Academy of Sciences of the United States of America.

[43] Luck, M. *et al*. A Photochromic Histidine Kinase Rhodopsin (HKR1) That Is Bimodally Switched by Ultraviolet and Blue Light. *Journal of Biological Chemistry* **287**, doi:10.1074/jbc.M112.401604 (2012). 10.1074/jbc.M112.401604 PMC3501036 23027869

[44] Schnedermann C (2016). Vibronic Dynamics of the Ultrafast all-trans to 13-cis Photoisomerization of Retinal in Channelrhodopsin-1. Journal of the American Chemical Society.

[45] Stensitzki T (2016). Femtosecond infrared spectroscopy of channelrhodopsin-1 chromophore isomerization. Struct Dyn.

[46] Stensitzki T, Muders V, Schlesinger R, Heberle J, Heyne K (2015). The primary photoreaction of channelrhodopsin-1: wavelength dependent photoreactions induced by ground-state heterogeneity. Front Mol Biosci.

[47] Hou SY (2012). Diversity of Chlamydomonas channelrhodopsins. Photochemistry and photobiology.

[48] Rost BR (2015). Optogenetic acidification of synaptic vesicles and lysosomes. Nature neuroscience.

[49] Zimanyi L, Saltiel J, Brown LS, Lanyi JK (2006). A priori resolution of the intermediate spectra in the bacteriorhodopsin photocycle: the time evolution of the L spectrum revealed. The journal of physical chemistry. A.

[50] Inoue K (2013). A light-driven sodium ion pump in marine bacteria. Nature communications.

[51] Hontani Y (2016). The photochemistry of sodium ion pump rhodopsin observed by watermarked femto- to submillisecond stimulated Raman spectroscopy. Physical chemistry chemical physics: PCCP.

[52] Alexandre MT (2009). Primary reactions of the LOV2 domain of phototropin studied with ultrafast mid-infrared spectroscopy and quantum chemistry. Biophysical journal.

[53] Ravensbergen J (2014). Unraveling the Carrier Dynamics of BiVO4: A Femtosecond to Microsecond Transient Absorption Study. The Journal of Physical Chemistry C.

[54] Mathes T (2015). Femto- to Microsecond Photodynarnics of an Unusual Bacteriophytochrome. Journal of Physical Chemistry Letters.

[55] Mathes T (2015). Proton-Coupled Electron Transfer Constitutes the Photoactivation Mechanism of the Plant Photoreceptor UVR8. Journal of the American Chemical Society.

[56] Kennis JTM, Groot M-L (2007). Ultrafast spectroscopy of biological photoreceptors. Current Opinion in Structural Biology.

[57] Papagiannakis E, Kennis JTM, van Stokkum IHM, Cogdell RJ, van Grondelle R (2002). An alternative carotenoid-to-bacteriochlorophyll energy transfer pathway in photosynthetic light harvesting. Proceedings of the National Academy of Sciences of the United States of America.

[58] Bonetti C (2010). Identification of excited-state energy transfer and relaxation pathways in the peridinin-chlorophyll complex: an ultrafast mid-infrared study. Physical Chemistry Chemical Physics.

[59] Snellenburg JJ, Laptenok SP, Seger R, Mullen KM, van Stokkum IHM (2012). Glotaran: A Java-Based Graphical User Interface for the R Package TIMP. J Stat Softw.

[60] Schapiro I, Weingart O, Buss V (2009). Bicycle-pedal isomerization in a rhodopsin chromophore model. Journal of the American Chemical Society.

[61] Blanco-Lomas, M., Campos, P. J. & Sampedro, D. Synthesis and Photoisomerization of Rhodopsin-Based Molecular Switches. *European Journal of Organic Chemistry*, 6328-6334 (2012).

[62] Vukovic L, Burmeister CF, Kral P, Groenhof G (2013). Control Mechanisms of Photoisomerization in Protonated Schiff Bases. Journal of Physical Chemistry Letters.

[63] Finley J, Malmqvist PA, Roos BO, Serrano-Andres L (1998). The multi-state CASPT2 method. Chemical Physics Letters.

[64] MacKerell AD (1998). All-atom empirical potential for molecular modeling and dynamics studies of proteins. Journal of Physical Chemistry B.

[65] Jorgensen WL, Chandrasekhar J, Madura JD, Impey RW, Klein ML (1983). Comparison of Simple Potential Functions for Simulating Liquid Water. Journal of Chemical Physics.

[66] Watanabe HC, Welke K, Sindhikara DJ, Hegemann P, Elstner M (2013). Towards an understanding of channelrhodopsin function: simulations lead to novel insights of the channel mechanism. J Mol Biol.

[67] Aquilante F (2010). Software News and Update MOLCAS 7: The Next Generation. J Comput Chem.

[68] Ponder JW, Richards FM (1987). An Efficient Newton-Like Method for Molecular Mechanics Energy Minimization of Large Molecules. J Comput Chem.

[69] Kloz M, Weissenborn J, Polivka T, Frank HA, Kennis JTM (2016). Spectral watermarking in femtosecond stimulated Raman spectroscopy: resolving the nature of the carotenoid S-star state. Physical Chemistry Chemical Physics.

