# Supplementary material for: Reaction dynamics of the chimeric channelrhodopsin C1C2
Source: Sci Rep. 2017 Aug 3;7:7217. doi: 10.1038/s41598-017-07363-w (PMC5543136; doi:10.1038/s41598-017-07363-w)
Supplement: Supplementary file 1 — supplementary information [file 41598_2017_7363_MOESM1_ESM.pdf]

Supplementary Information

Reaction dynamics of the chimeric channelrhodopsin C1C2

Yusaku Hontani[†], Marco Marazzi[‡,§,¶], Katja Stehfest[#], Tilo Mathes[†], Ivo H.M. van Stokkum[†], Marcus Elstner[‡], Peter Hegemann[#] and John T. M. Kennis[†*]

[†]*Department of Physics and Astronomy, Vrije Universiteit Amsterdam, De Boelelaan 1081, 1081 HV Amsterdam, The Netherlands*

[‡]*Institute of Physical Chemistry, Karlsruhe Institute of Technology, Karlstrasse 12, D-76131 Karlsruhe, Germany*

[§]*Unidad Docente de Química Física, Universidad de Alcalá, E-28871 Alcalá de Henares, Madrid, Spain*

[#]*Institute of Biology, Experimental Biophysics, Humboldt-Universität, Invalidenstraße 42, D-10115 Berlin, Germany*

*Corresponding author: j.t.m.kennis@vu.nl

[¶]Present Address: Théorie-Modélisation-Simulation, Université de Lorraine-Nancy & CNRS, SRSMC, Boulevard des Aiguillettes, Vandoeuvre-lès-Nancy, France

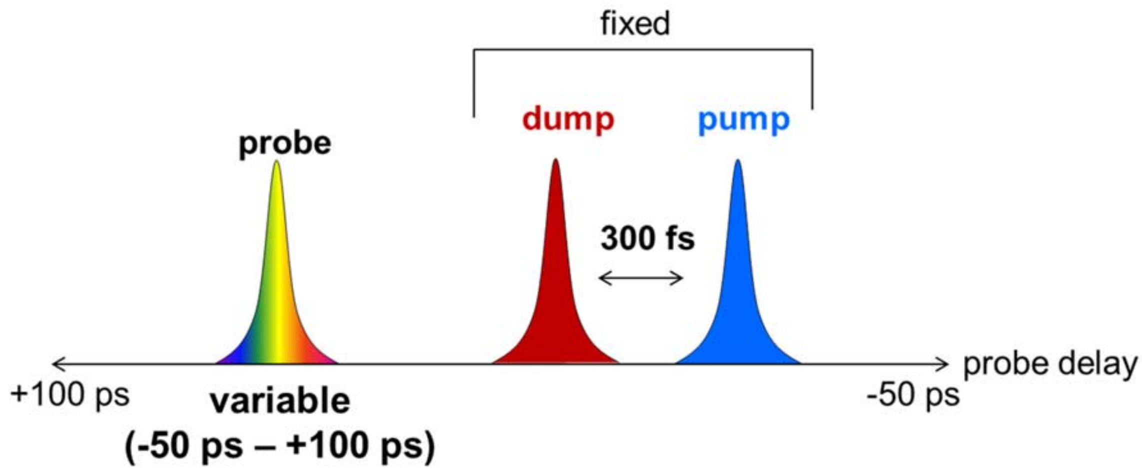

Figure S1. Scheme of pump-dump-probe experiments. Wavelengths of pump, dump and probe pulses were 480 nm, 720 nm and 430–750 nm, respectively. Time difference between the pump and dump pulses was fixed as 300 fs. Time difference between the pump and probe pulses was valuable from -50 ps to +100 ps.

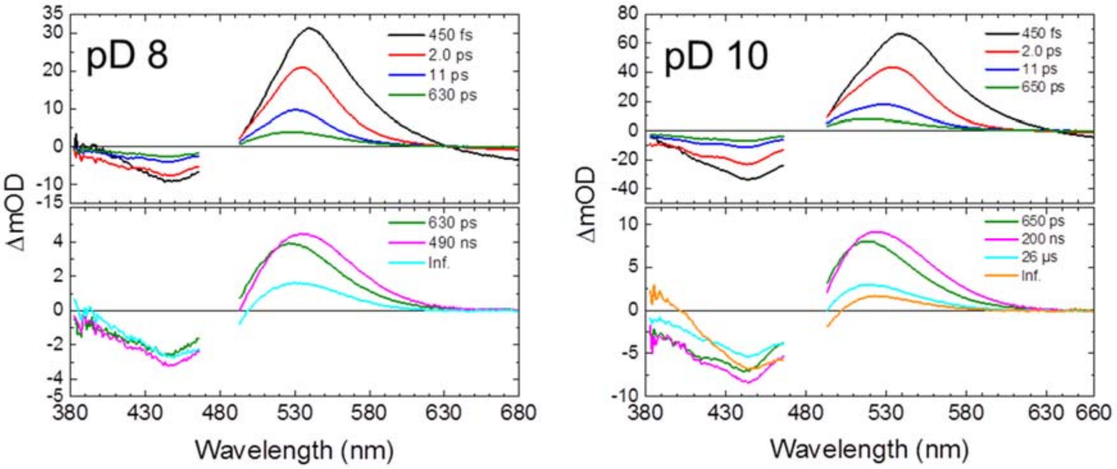

Figure S2. Evolution-associated difference spectra (EADS) and Decay-associated difference spectra (DADS) of C1C2 at pD 8 and 10. The spectral region of 465–495 nm is omitted because of the strong pump scattering. The 630 ps (pD 8) and 650 ps (pD 10) components are shown on both of the top and the bottom figures for reference.

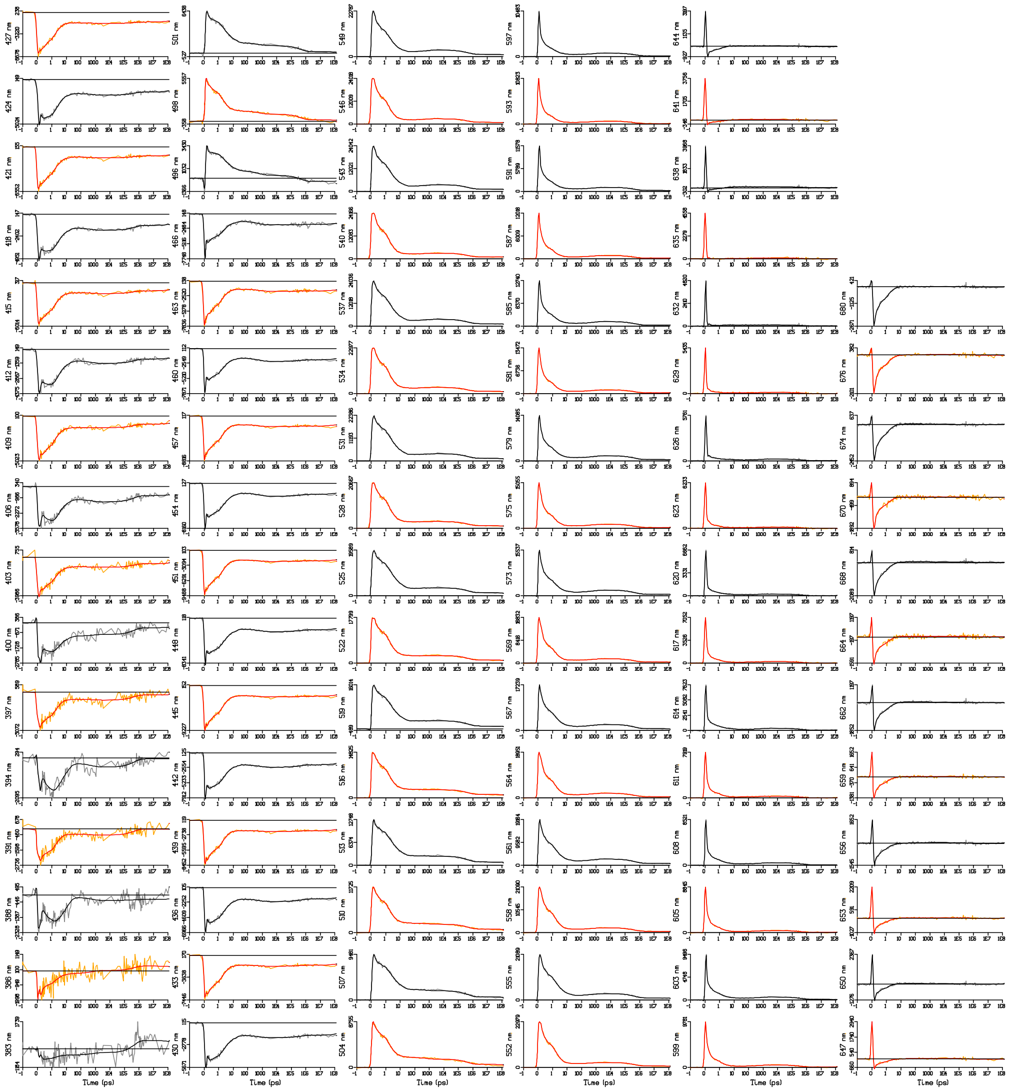

Figure S3. Selected time traces of fs-µs pump-probe experiments in C1C2 at pH 8 and pD8. pH 8 data (grey, in mOD), pD8 data (orange), and fit (respectively, black, and red) are shown at selected wavelengths. Wavelength is written in the ordinate label. Note that the time axis is linear until 1 ps (after the maximum of the IRF), and logarithmic thereafter.

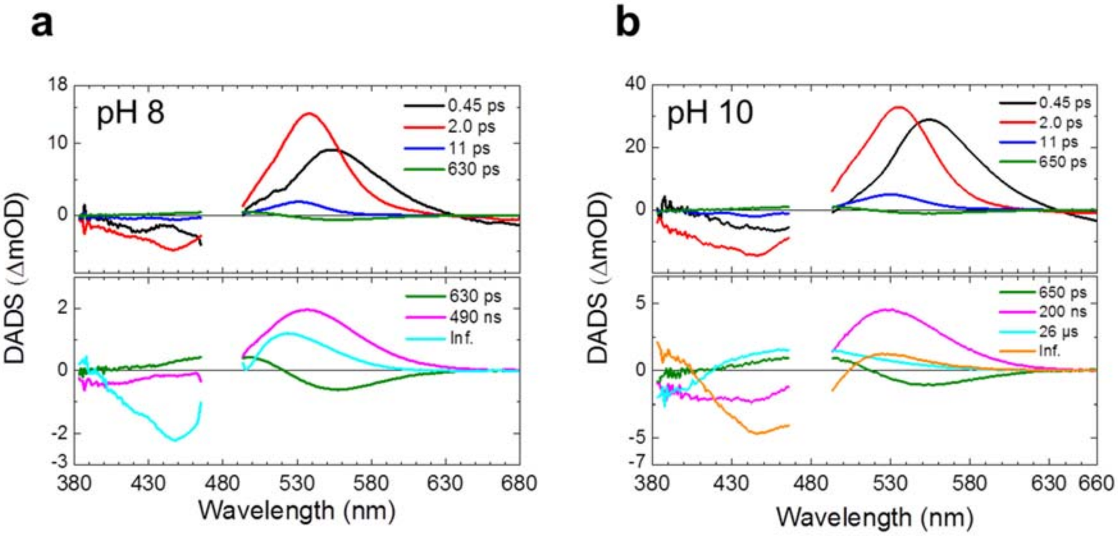

Figure S4. Decay-associated difference spectra (DADS) of C1C2 at (a) pH 8 and (b) pH 10. The spectral region of 465–495 nm is omitted because of the strong pump scattering. The 630 ps (pH 8) and 650 ps (pH 10) components are shown on both of the top and the bottom figures for reference.

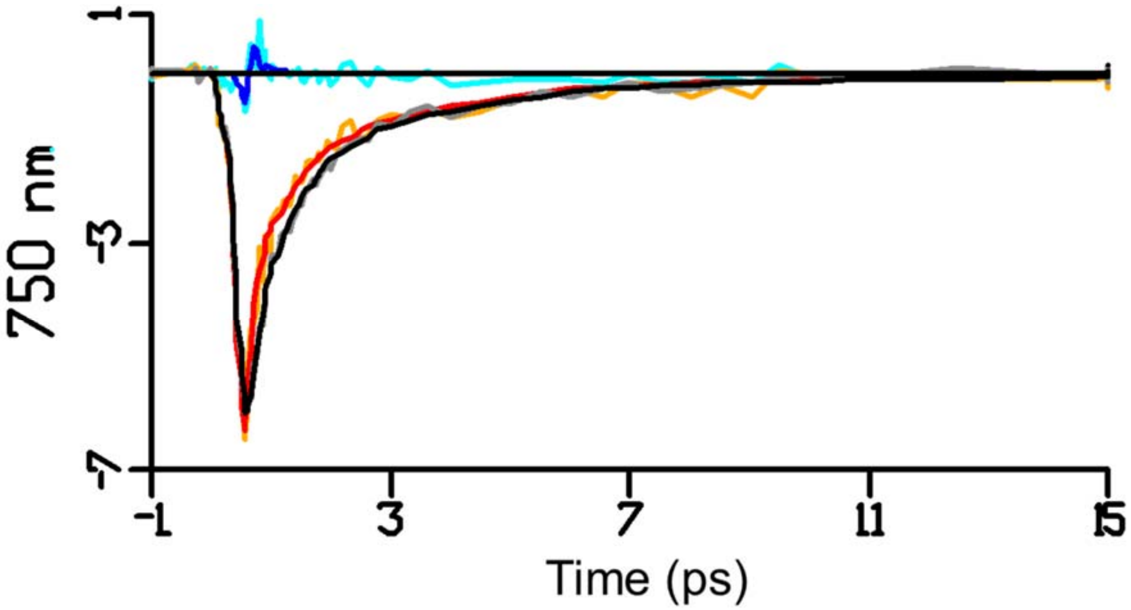

Figure S5. Time trace of pump-probe and pump-dump probe experiments in C1C2 at pH 8 at 750 nm. Pump-probe data (grey, in mOD), pump-dump-probe data (orange), dump-probe data (cyan) and fit (respectively, black, red and blue) are shown at selected wavelengths. Note that the time axis is linear, and that only are shown from -1 to 15 ps (after the maximum of the IRF).

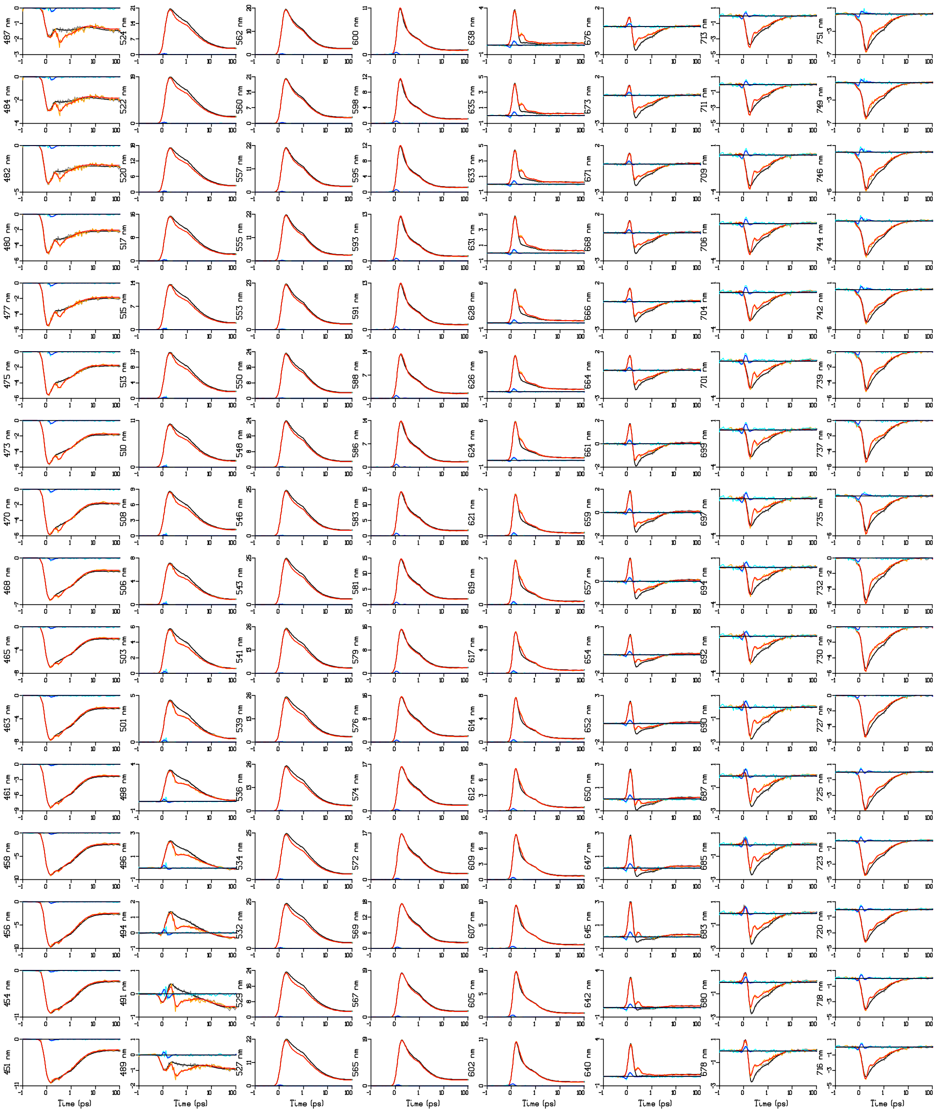

Figure S6. Selected time traces of pump-dump-probe experiments in C1C2 at pH 8. Pump-probe data (grey, in mOD), pump-dump-probe data (orange), dump-probe data (cyan) and fit (respectively, black, red and blue) are shown at selected wavelengths. Wavelength is written in the ordinate label. Note that the time axis is linear until 1 ps (after the maximum of the IRF), and logarithmic thereafter.

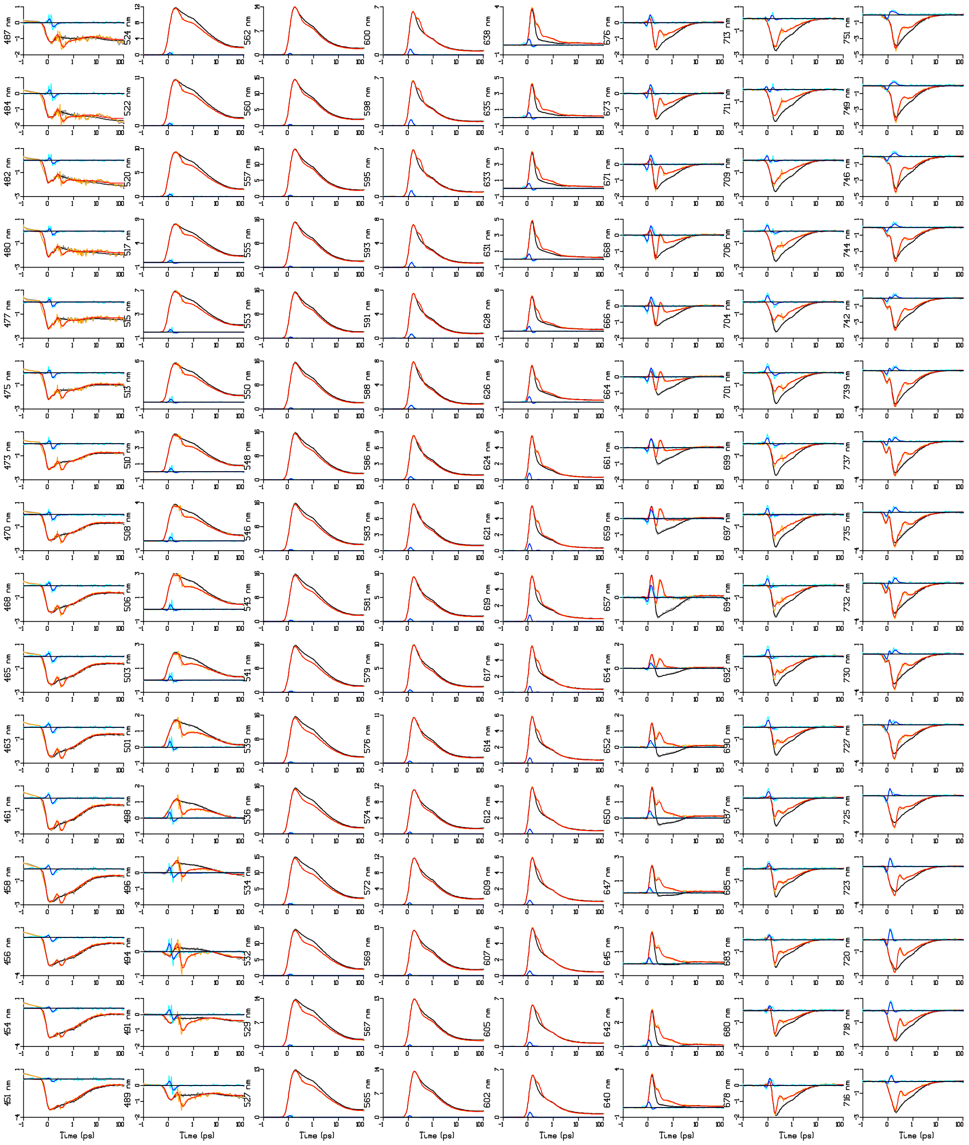

Figure S7. Selected time traces of pump-dump-probe experiments in C1C2 at pD 8. Pump-probe data (grey, in mOD), pump-dump-probe data (orange), dump-probe data (cyan) and fit (respectively, black, red and blue) are shown at selected wavelengths. Wavelength is written in the ordinate label. Note that the time axis is linear until 1 ps (after the maximum of the IRF), and logarithmic thereafter.

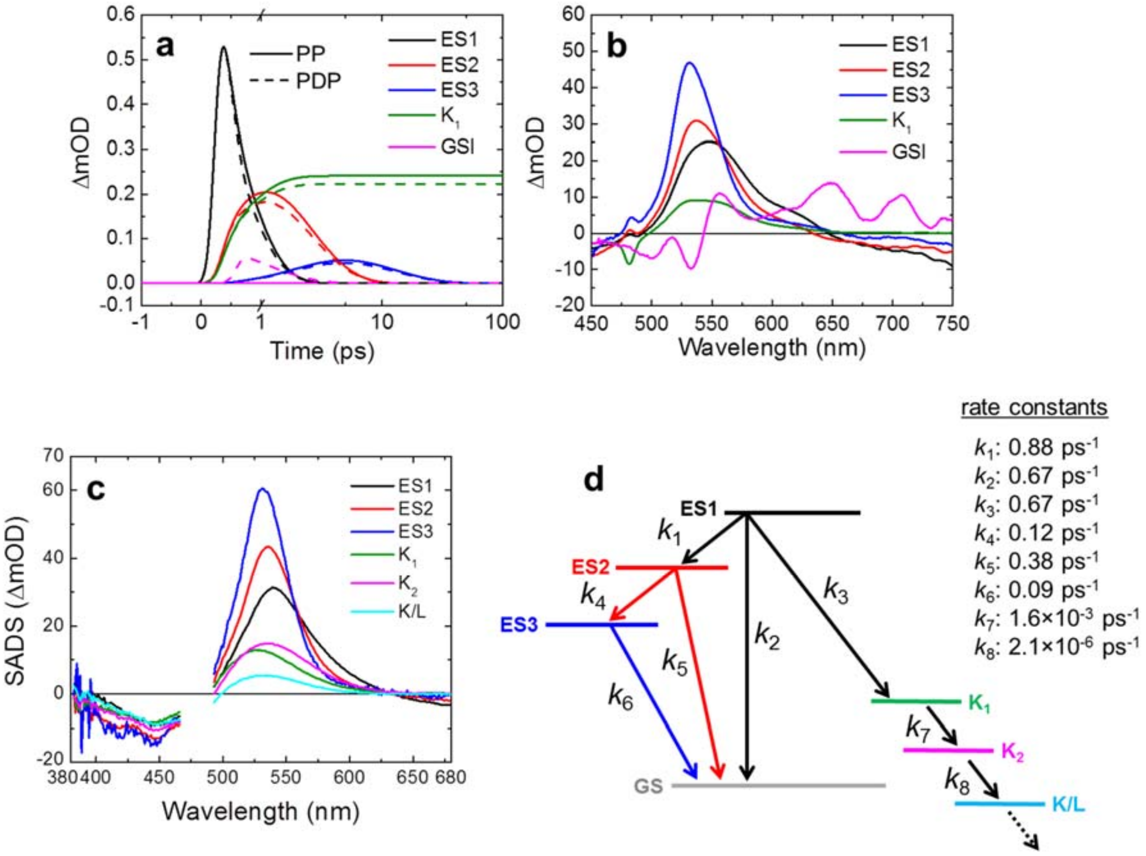

Figure S8. Target analysis for time-resolved experiments for C1C2. a,b, Target analysis for pump-dump-probe experiments at pD 8. **a,** Time evolution of population rate of three excited states (ES1, ES2 and ES3), the first photoproduct state (K_1) and ground state intermediate (GSI). The target model of Fig. 3b in the main text was applied. Solid and dashed lines show evolution of pump-probe (PP) and pump-dump-probe (PDP) data, respectively. The time axis is linear until 1 ps, and logarithmic thereafter. **b,** Species-associated difference spectra (SADS) of pump-dump-probe experiments. **c,d,** Target analysis for femto- to submillisecond pump-probe data of C1C2 at pH 8. **c,** SADS of pump-probe experiments at pH 8. The same spectroscopic data as Fig. 2a in the main text was used. The spectral region of 465–495 nm is omitted because of the strong pump scattering. **d,** Kinetic scheme consisting of three excited states (ES1, ES2 and ES3), three photoproduct state (K_1, K_2 and K/L).

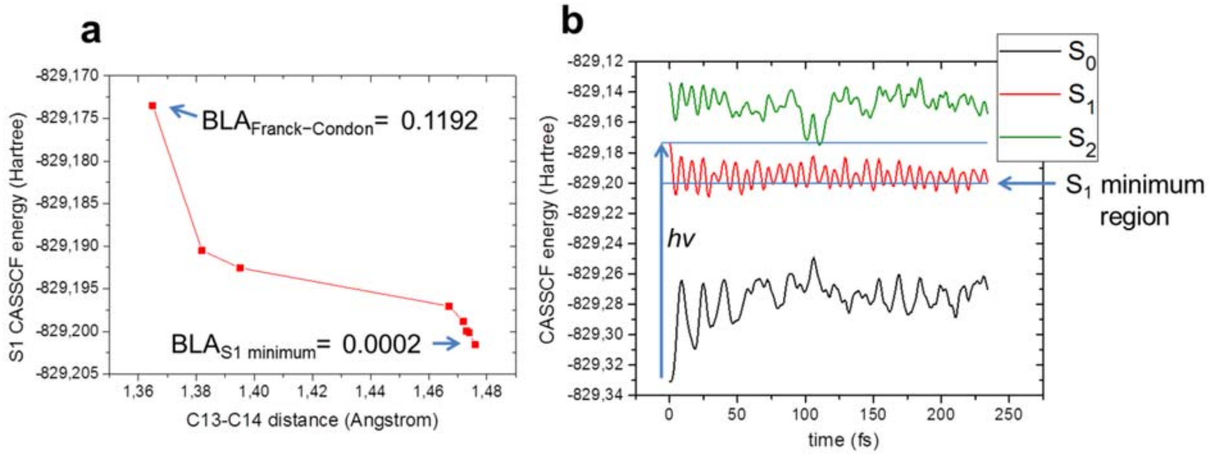

Figure S9. Computational study from Franck−Condon to the S_1 minimum region, for the retinal chromophore hydrogen bonded to deprotonated Glu162. (a) S_1 minimum energy path, plotted as CASSCF energy vs. C13-C14 distance: a partial inversion of the bond order – from double to single – is evidenced also from the bond length alternation (BLA) calculated for the initial and final geometries of the path. (b) S_1 excited state dynamics showing the initial vibrational relaxation of the retinal, up reaching the S_1 minimum region.

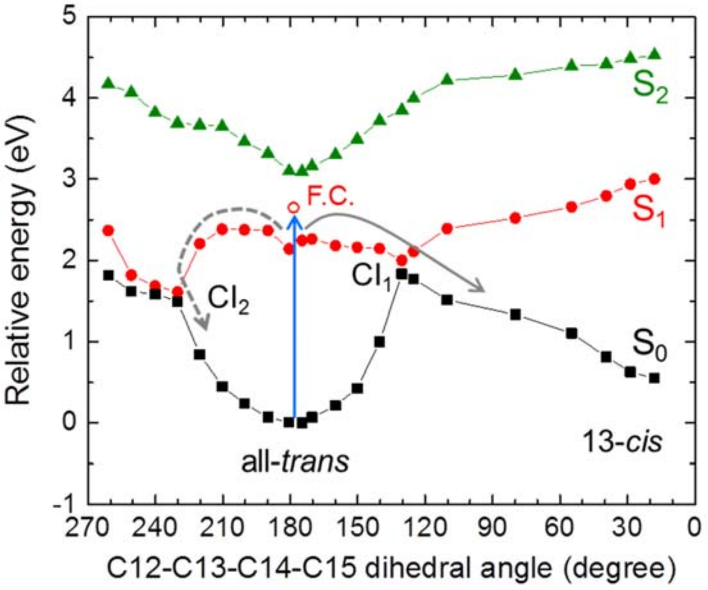

Figure S10. CASPT2//CASSCF(12,12) photoisomerization pathways from the all-*trans* retinal conformer, when the Schiff base is hydrogen-bonded to a water molecule W2. S_0 (black closed squires with black solid line), S_1 (red closed dots with red solid line) and S_2 (green closed triangles with green solid line) energy profiles are shown. The blue arrow shows light excitation of all-*trans* retinal as performed on our pump-probe and pump-dump-probe spectroscopic experiments. The open red dot shows the Franck-Condon state. The solid gray arrow shows the isomerization path through energy barrier of 2.10 kcal/mol (0.09 eV) on S_1 via a conical intersection CI_1. The dashed gray arrow indicates a relaxation path through energy barrier of 5.20 kcal/mol (0.23 eV) on S_1 via another conical intersection CI_2.

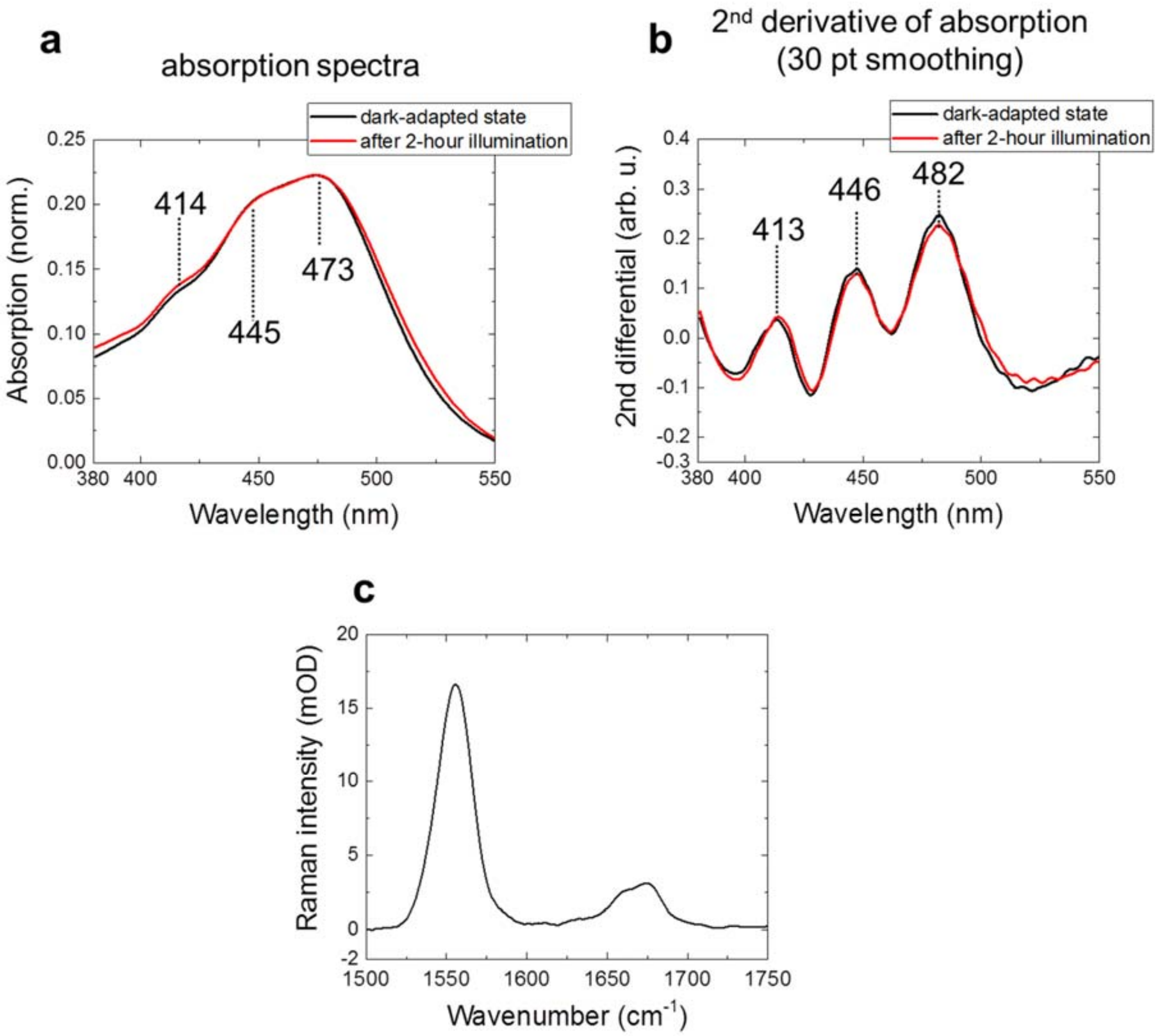

Figure S11. Steady-state absorption and stimulated Raman spectra of C1C2 at pH 8. a, UV-Vis absorption spectra and **b,** 2nd derivatives of the absorption spectra (30-point Savitzky-Golay smoothing was applied) of C1C2 at pH 8, taken with the same experimental condition as transient absorption experiments on a Lissajous scanner. Spectra of dark-adapted state and after 2-hour illumination with 0.2 mW femtosecond laser (500 Hz, 480 nm, spot size: ~200 μm) are shown in black and red lines, respectively. **c,** stimulated Raman spectra of dark-state C1C2 at pH 8 spectra (10-point Savitzky-Golay smoothing and baseline correction were applied).

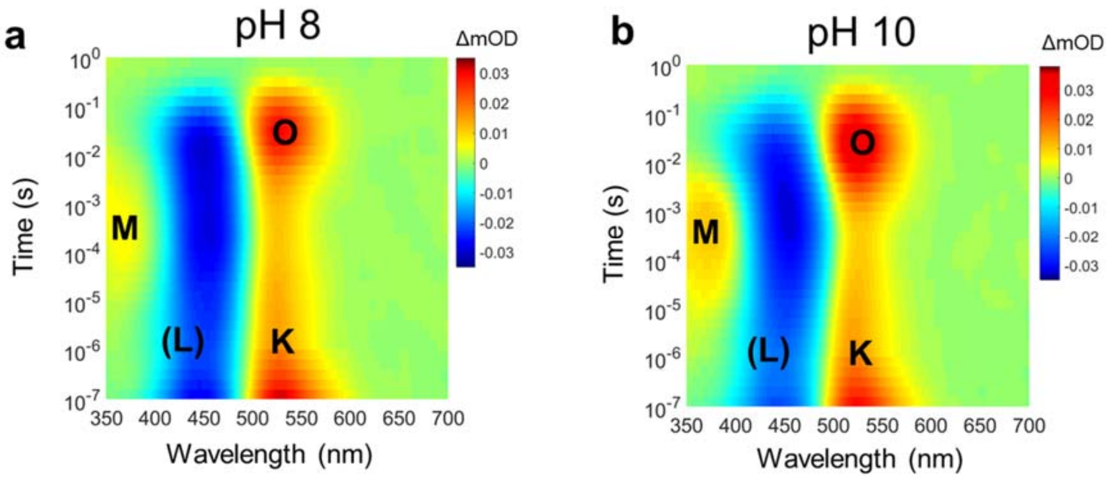

Figure S12. Counterplots of flash photolysis experiments for microsecond-to-second kinetics of C1C2 at pH 8 and pH 10.

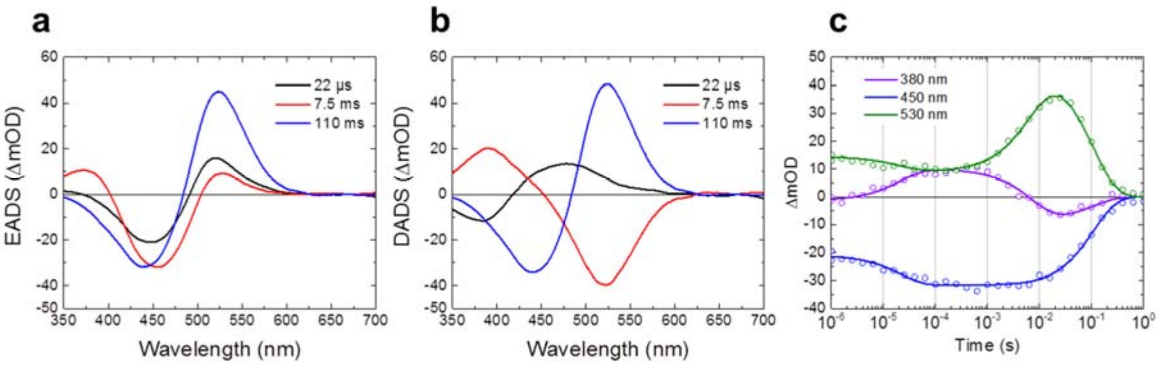

Figure S13. Globally fitted spectra and selected time traces of flash photolysis experiments in C1C2 at pH 10. (a) EADS, **(b)** DADS and **(c)** time traces with fitting curves (solid lines) at 380 (purple), 450 (blue) and 530 nm (green).

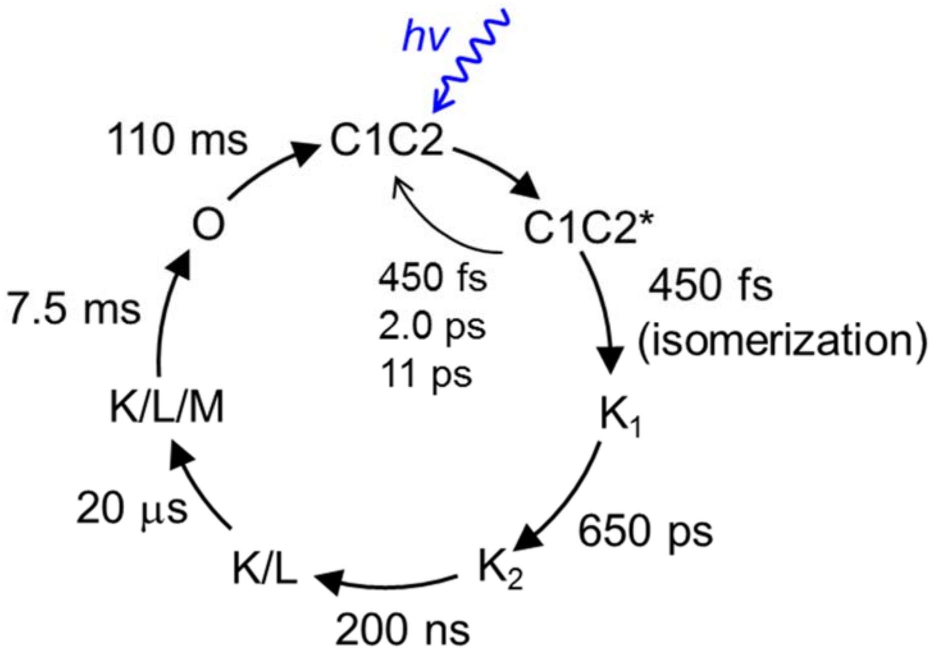

Figure S14. Photocycle models of C1C2 at pH 10.
